# Driving forces of soil bacterial community structure, diversity, and function in temperate grasslands and forests

**DOI:** 10.1038/srep33696

**Published:** 2016-09-21

**Authors:** Kristin Kaiser, Bernd Wemheuer, Vera Korolkow, Franziska Wemheuer, Heiko Nacke, Ingo Schöning, Marion Schrumpf, Rolf Daniel

**Affiliations:** 1 Department of Genomic and Applied Microbiology, Institute of Microbiology and Genetics, Georg-August-University Göttingen, Grisebachstr. 8, D-37077 Göttingen, Germany; 2 Department of Crop Sciences, Georg-August-University Göttingen, Grisebachstr. 6, D-37077 Göttingen, Germany; 3 Max Planck Institute for Biogeochemistry, Hans-Knoell-Str. 10, D-07745 Jena, Germany.

## Abstract

Soil bacteria provide a large range of ecosystem services such as nutrient cycling. Despite their important role in soil systems, compositional and functional responses of bacterial communities to different land use and management regimes are not fully understood. Here, we assessed soil bacterial communities in 150 forest and 150 grassland soils derived from three German regions by pyrotag sequencing of 16S rRNA genes. Land use type (forest and grassland) and soil edaphic properties strongly affected bacterial community structure and function, whereas management regime had a minor effect. In addition, a separation of soil bacterial communities by sampling region was encountered. Soil pH was the best predictor for bacterial community structure, diversity and function. The application of multinomial log-linear models revealed distinct responses of abundant bacterial groups towards pH. Predicted functional profiles revealed that differences in land use not only select for distinct bacterial populations but also for specific functional traits. The combination of 16S rRNA data and corresponding functional profiles provided comprehensive insights into compositional and functional adaptations to changing environmental conditions associated with differences in land use and management.

Soil bacteria play an important role in biogeochemical cycles^1^,^2^. They control soil processes such as decomposition^3^ and mineralization, including the associated release of greenhouse gases such as carbon dioxide (CO_2_), nitrous oxide (N_2_O), and methane (CH_4_)^4^,^5^ into the atmosphere. Moreover, several soil bacteria promote plant growth and productivity^2^,^6^. As soil represents a highly dynamic and complex environment, bacterial communities living in this ecosystem are influenced by a multitude of different biotic and abiotic factors. Previous studies showed that soil pH is a major driver of these communities^7^,^8^,^9^. Lauber and colleagues^8^ observed that the overall bacterial community composition in different soils from across South and North America was significantly correlated with soil pH. This was confirmed by a study of bacterial communities in German grassland and forest soils^9^. Other studies investigating the effect of edaphic parameters on soil bacteria found that these communities were influenced by the availability of nutrients such as carbon, nitrogen^10^,^11^, and soil moisture in grasslands^12^ and forests^13^.

In recent years, the impact of land use intensification on bacterial community diversity and composition, e.g. by fertilization in grasslands, has been frequently investigated^14^,^15^,^16^,^17^. In a study by Herzog *et al.*^15^, composition and diversity of entire and active bacterial communities were altered by fertilizer application. Lauber *et al.*^16^ analyzed soil bacterial communities across different land use types such as grasslands and forests. For soil bacteria in forest systems, soil disturbance and organic matter removal^18^,^19^ as well as the dominant tree species^20^ have been shown to influence community composition. This provides evidence that land use intensification can alter soil bacterial community composition. However, most studies have focused on a limited number of soil samples in one region. Therefore, the response of bacterial communities in grasslands and forests to land use intensification and environmental changes is not yet fully understood. Large comparative studies are required to unravel the diverse interactions between bacteria and their environments, and how changes in community composition might reflect changes in bacterial functioning.

The aim of the present study was to identify key drivers of bacterial community composition, diversity, and functions in forest and grassland soils. In addition, we aimed at clarifying in which way soil bacterial communities respond to management regime, and if changes are merely a product of the edaphic properties. In this study, 300 soil samples were taken from the three German Biodiversity Exploratories Schorfheide-Chorin, Hainich-Dün and the Schwäbische Alb^21^. Two previous studies focusing on subsets of samples taken in the Biodiversity Exploratories showed that bacterial diversity was influenced by land use intensity^22^ and land use type^9^. Bacterial communities were assessed by pyrotag sequencing targeting the bacterial 16S rRNA gene. Additionally, functional profiles were calculated from obtained 16S rRNA gene data^23^. We focused on three main hypotheses: (1) soil bacterial communities exhibit distinct biogeographic patterns, (2) respond differently to soil conditions and land use intensification, and (3) bacterial community composition, diversity and functioning are shaped in a similar way within the same land use system.

## Results and Discussion

### General characteristics of the soil samples

Soil samples showed significant differences with respect to soil texture and edaphic properties (Table 1, Supplementary Material Tables S1 and S2). Forest soils were more acidic, had a higher C:N ratio and smaller clay amount than grassland soils. Forest soil samples derived from the different exploratories exhibited significant differences in all measured edaphic properties. The Schorfheide-Chorin forest soils were more acid and had higher C:N ratios compared to the Hainich-Dün and Schwäbische Alb soils, which did not differ significantly. In addition, Schorfheide-Chorin forest soils also exhibited the lowest gravimetric water content, clay and silt amount of all exploratories.

Grassland soil samples derived from the different exploratories also exhibited significant differences between all measured edaphic properties. The Hainich-Dün grasslands soil had the highest pH values, lowest gravimetric water content and highest silt amount compared to the Schorfheide-Chorin and Schwäbische Alb soil, which did not differ significantly. The Schorfheide-Chorin grassland soils exhibited the highest C:N ratio and sand amount compared to the other two exploratories. Clay amount was lowest in the Schorfheide-Chorin grassland soils, followed by the Hainich-Dün soils. The highest clay amounts were determined for the Schwäbische Alb grassland soils. Significant differences in soil parameters between the different management regimes were not recorded (ANOVA, P > 0.5 in all cases).

### Soil bacterial communities

Composition and diversity of soil bacterial communities were assessed by pyrotag sequencing of 16S rRNA genes. After quality filtering, denoising, and removal of potential chimeras and non-bacterial sequences, approximately 2,700,000 high quality sequences with an average read length of 525 bp were obtained for further analyses. All sequences were classified below phylum level. Based on richness estimator data (Michaelis-Menten fit; Supplementary Material Table S3) 78–88% of the operational taxonomic units (OTUs) at 80% identity (phylum level) and 27–55% of the OTUs at 97% identity (species level) were covered by the surveying effort (for rarefaction curves, see Supplementary Material Figs S1 and S2).

**Figure 1:**
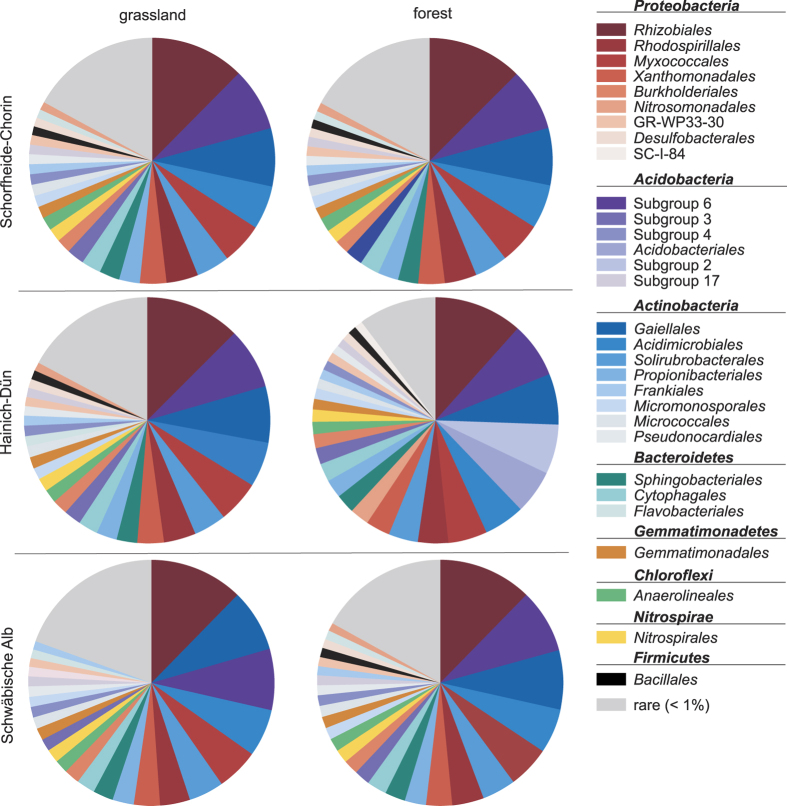
Abundances of bacterial orders in Schorfheide-Chorin, Hainich-Dün and Schwäbische Alb grassland and forest soils. Mean abundances of the most abundant bacterial orders (>1% of the total bacterial community) for each exploratory and land use are given. Rare: sum of bacterial orders contributing <1% to the total bacterial community per exploratory.

Obtained sequences clustered into 203,530 OTUs (97% identity) and were assigned to 51 bacterial phyla, 574 orders and 1,215 families. The dominant phyla and proteobacterial classes (>1% of all sequences across all samples) were *Actinobacteria* (23.75% ± 8.55%), *Alphaproteobacteria* (20.43% ± 5.21%), *Acidobacteria* (18.39%% ± 9.19%), *Deltaproteobacteria* (7.22% ± 2.84%). *Bacteroidetes* (5.15% ± 2.60%), *Chloroflexi* (5.09% ± 2.10%), *Betaproteobacteria* (4.64% ± 2.38%), *Gammaproteobacteria* (4.32% ± 1.23%), *Gemmatimonadetes* (1.88% ± 0.92%), *Firmicutes* (1.18% ± 3.20%), and *Nitrospirae* (1.14% ± 1.10%). These phylogenetic groups were present in all samples and accounted for more than 95% of all sequences analyzed in this study (Fig. 1). These results are consistent with previous studies on grasslands^24^ and temperate beech forests^25^. The most abundant phylotype (3.99% ± 2.44) is an uncultured member of the Subgroup 6 of the *Acidobacteria*. The five most abundant phylotypes that could be assigned to a genus are *Bradyrhizobium* (2.66% ± 1.45%), *Candidatus Solibacter* (2.00% ± 1.86%), *Haliangium* (1.39% ± 0.74%), *Variibacter* (1.36% ± 0.58%) and *Gaiella* (1.34% ± 1.31%) of all sequences, respectively.

### Biogeographic variations of soil bacterial diversity and community composition

Diversity (represented by the Shannon index H’) and community structure of soil bacteria (PERMANOVA, P < 0.001) differed between the three Biodiversity Exploratories. The Hainich-Dün exploratory harbored the most diverse bacterial community (H’ = 10.22) compared to Schorfheide-Chorin (H’ = 9.72) and the Schwäbische Alb (H’ = 9.92). Furthermore, grassland soils are significantly more diverse than forest soils (H’ = 10.12 and H’ = 9.48, respectively, with P < 0.001), which supports previous findings of Nacke and colleagues^9^, who reported that bacterial communities were more diverse in grasslands at phylum level. As samples derived from forests soils were more acidic than grassland soil samples (P < 0.001), the difference in pH might explain the difference in diversity (Table 1).

The most dominant bacterial orders of the complete dataset differed in their distribution across the three exploratories. These differences most likely arose from differrences in edaphic properties in the exploratories. Therefore, we tested for correlation of environmental factors by NMDS analysis based on Bray-Curtis dissimilarities. Fitting the edaphic properties to the ordination revealed the pH as the strongest driver of the community. Additional canonical correspondence analysis (CCA) using pH as constrain showed that pH explains 26% of the variation in community structure (P < 0.001, Supplementary Figure S3). We additionally found a separation of soil bacterial communities by sampling region (PERMANOVA, P < 0.001) and the two land use types grassland and forest (PERMANOVA, P < 0.001) (Supplementary Figure S4). Therefore, we were interested in a detailed analysis of the factors driving the changes in the structure of bacterial communities in each exploratory. We further split the data between grasslands and forests due to the strong separation between the community structure of both land use types.

### Key drivers of bacterial communities

**Figure 2:**
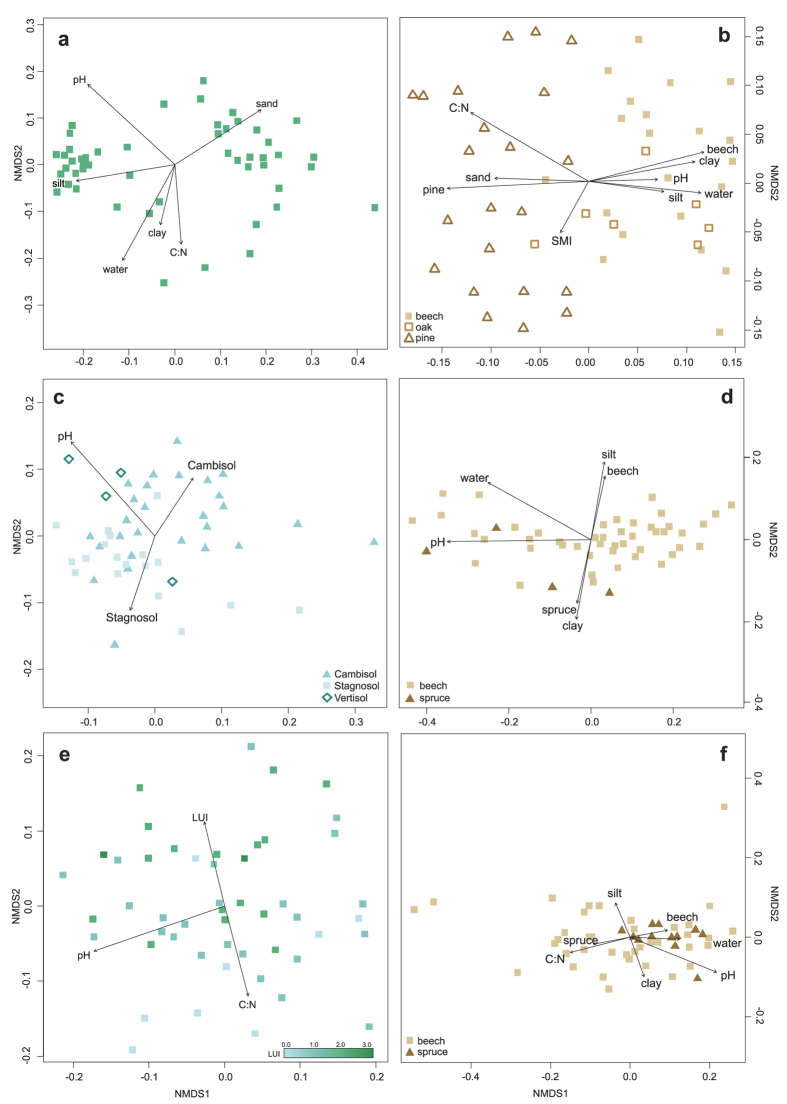
NMDS plots split by region and land use. NMDS plots based on Bray Curtis dissimilarities of grassland (**a,c,e**) and forest (**b,d,f**) bacterial communities. Environmental parameters that are significantly (P < 0.05) correlated are indicated as arrows (C:N: carbon: nitrogen ratio; water: gravimetric water content; sand: sand amount; silt: silt amount; clay: clay amount; LUI: land use intensity index in grasslands; SMI: silvicultural management index in forests). (**a**) Schorfheide-Chorin grassland samples; (**b**) Schorfheide-Chorin forest samples; (**c**) Hainich-Dün grassland samples; (**d**) Hainich-Dün forest samples; (**e**) Schwäbische Alb grassland samples; (**f**) Schwäbische Alb forest samples. Note that the NMDS axes have different scales for each ordination.

To identify the key drivers of soil bacterial community structure for each land use type in each exploratory, we performed NMDS analysis for the six subsets. The soil pH was the only property, which affected the community structure in each subset (Fig. 2). Another property influencing the community structure in grasslands and forests was soil texture (amount of clay, sand and/or silt), which represents pore size, water and gas fluxes, and nutrient availability^26^,^27^. Moreover, soil texture is important for niche separation and protection from predation^28^.

In grassland soils, the C:N ratio influenced bacterial community structure in the Schwäbische Alb and Schorfheide-Chorin, but not in the Hainich-Dün. This is supported by a PFLA-based study on soil bacterial communities, in which edaphic properties such as soil texture, pH, and C and N concentration were involved in structuring soil bacterial communities^10^. The land use intensity index (LUI) was only correlated with the Schwäbische Alb grassland community. However, the LUI only accounts for the amount and not for the source of fertilization. In the Schwäbische Alb grasslands, most plots received organic fertilizer (manure, dung), whereas fertilization in the Hainich-Dün and Schorfheide-Chorin was predominated by mineral fertilizer application. These findings support a recent study, in which soil microbial communities of farming systems receiving organic fertilizer were different compared to those of conventional, minerally fertilized systems and control soils^29^. In agreement with Geisseler and Scow^30^, clear trends suggesting bacterial community structural shifts due to long-term mineral fertilizer application, were not found in our survey.

In forest soils, the tree species was correlated with bacterial community structure in all exploratories, while the silvicultural management index (SMI) only significantly influenced the community structure in the Schorfheide-Chorin (Fig. 2). Soil bacterial communities under broadleaved (*Fagus* and *Quercus*) and coniferous (*Pinus* and *Picea*) trees formed distinct patterns. This is in accordance with results of previous studies^9^,^20^. Nacke *et al.*^9^ analyzed a subset of soil samples derived from the Schwäbische Alb and found that the bacterial community structure was different under beech (*Fagus*) and spruce (*Picea*). This is consistent with a study comparing bacterial communities under coniferous and broadleaved trees^20^. We did not observe a difference between the two broadleaved tree species, although differences in soil community structure between broadleaved trees have been described for *Fagus* versus *Tilia* and *Acer*^31^. These effects might be partly due to the reduced soil acidification and higher turnover rates of the leaf litter of *Tilia* and *Acer*^32^. Coniferous tree species such as spruce (*Picea abies*) and pine (*Pinus sylvestris*) are known to significantly decrease the soil pH (reviewed in ref. 33) due to the special chemical structure of evergreen litter or capture of atmospheric acidic compounds^34^. This would result in an indirect pH effect on soil bacteria. Additionally, this might be one of the reasons why tree species play an important role in the structuring of bacterial communities in all forest samples analyzed.

According to our hypothesis that bacterial community structure and diversity would be affected in similar ways under the same land use, we compared the bacterial diversity, represented by the Shannon index (H’), between the different management regimes (Supplementary Material Table S4). Differences in diversity were detected for the tree species in the Schwäbische Alb and Schorfheide-Chorin.

Interestingly, the management regimes in grasslands (meadow, pasture, mown pasture) and forests (unmanaged forest, age-lass forest, selection forest) exhibited no significant effect on bacterial diversity (PERMANOVA, P < 0.05). This is in contrast to a previous study by Will *et al.*^22^, who found a higher bacterial diversity in grassland soils of low land use intensity in the Hainich-Dün. In contrast, Tardy *et al.*^17^ investigated bacterial diversity along gradients of land use intensity and observed the highest bacterial diversity in moderately managed soils. The authors suggest that this effect is related to the stress response of the bacterial community. In highly stressed environments, as under high land use intensity, diversity decreases due to the dominance of competitive species and competitive exclusion, while in unstressed environments diversity decreases due to the dominance of adapted species through selection. In accordance with our hypothesis, we could find soil conditions such as pH that consistently drive bacterial community structure as well as diversity, while management regimes and therefore land use intensity have no significant influence. In addition, we could show that pH is the best predictor of bacterial communities.

### Bacterial functioning in grassland and forest soils

**Figure 3:**
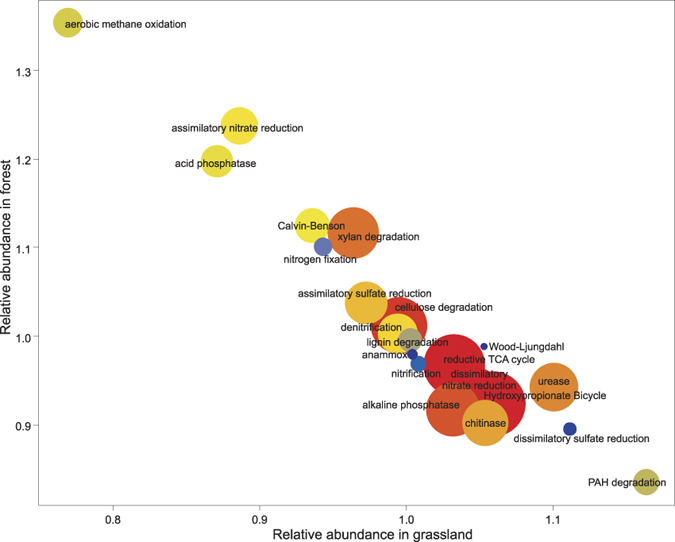
Relative abundances of key enzymes in grassland and forest. Key genes for nitrogen, sulfur, and methane metabolism, carbon fixation pathways, cellulose, xylan, lignin and polyaromatic-hydrocarbon (PAH) degradation, acid and alkaline phosphatases and urease were combined. Their mean abundance (relative to the mean in the complete dataset) in grasslands soil was plotted against the mean abundance in forest soils. Size and color of the circles indicate the mean abundance in the complete dataset. Low abundance: small blue circles; medium abundance: medium yellow circles; high abundance: large red circles. The enzymes included in the analysis are given in Supplementary Material Table S5.

We further hypothesized that bacterial functioning was driven in a similar manner as bacterial community structure and diversity. To clarify this hypothesis, we focused on pathways involved in the cycling of carbon, nitrogen, phosphorus, and sulfur (Fig. 3) and compared the relative abundances of key enzyme-encoding genes between the two land uses grassland and forest. Abundances of the enzyme-encoding genes were derived from a novel bioinformatic tool Taxa4Fun^23^. Tax4Fun transforms the SILVA-based OTUs into a taxonomic profile of KEGG organisms, which is normalized by the 16S rRNA copy number (obtained from NCBI genome annotations). As soils harbor unknown or uncultured organisms, not all 16S sequences can be mapped to KEGG organisms. Spearman correlation analysis of functional profiles derived from whole metagenome sequencing and profiles deduced from 16S rRNA gene sequences revealed a median of the correlation coefficient of 0.8706 for soils^23^. This indicated that Tax4Fun provides a good approximation to functional profiles obtained from metagenomic shotgun sequencing approaches. This is especially valuable to deduce functional profiles for a large number of samples derived from complex environments, as achieving representative coverage for each sample of a large sample set by metagenome shotgun sequencing would be a daunting task.

Most key enzyme-encoding genes involved in the cycling of C, N, S, and P are either more abundant in grassland or forest soils (Mann-Whitney test, P < 0.05, Supplementary Material Table S5). For example, genes that encode acid phosphatases were observed at 1.4-fold higher abundances in the functional profile of the forest soils than in the grassland soils, while alkaline phosphatases showed the opposite trend. We assume that this effect could be attributed to the difference in pH between the land use types, as we showed that pH is the best predictor for bacterial communities. The genes encoding urease were 1.2-fold more abundant in the grassland. The availability of urea was higher in the grassland samples, as these are partly fertilized with manure or dung or were grazed by animals. Chitinase genes also showed a 1.2-fold higher abundance in grasslands compared to forest soils. This might result from the higher abundance of *Actinobacteria* in grassland soils, as this group is known to harbor a high number of chitinase genes^35^. Genes involved in polyaromatic hydrocarbon (PAH, here lignin) degradation are more abundant in grasslands. In forest systems, this process is primarily performed by ligninolytic fungi (mainly saprotrophic basidiomycetes), which are able to degrade wooden biomass^36^. One key enzyme for aerobic methane oxidation, methanol dehydrogenase, was notably more abundant in forest soils. Methane oxidation in forest soils is the largest biological sink for atmospheric methane^4^ and therefore plays a critical role in the flux of this greenhouse gas. Additionally, nitrous-oxide (N_2_O) reductase, which catalyzes the last step in denitrification and reduces N_2_O to N_2_, is also more abundant in forest soils (data not shown). These results indicate that temperate forest ecosystems not only play a crucial role in the regulation and removal of methane, but also of the greenhouse gas nitrous oxide.

Interestingly, the key enzyme of nitrogen fixation, the nitrogenase, is less abundant in grassland than in forest soils. In this study, only bulk soil was sampled and therefore presumably only free-living nitrogen-fixing bacteria could be detected. It is possible, that nitrogen fixation by free-living bacteria plays a greater role in forest systems, whereas symbiotic and rhizospheric bacteria, which were not covered by the study, carry out the major part of nitrogen fixation in grassland systems.

The obtained results suggest that the different land uses grassland and forest not only select for distinct bacterial populations, but also for specific functional traits within their bacterial communities. As the grasslands and forests analyzed in the present study are long-term established systems, it would be interesting to evaluate if a similar adaptation is also present in younger systems.

### Soil pH is the best predictor of bacterial communities

In the present study, pH was the only factor, which influenced the bacterial community regardless of exploratory and land use. Furthermore, it not only affected bacterial community structure, but also the functional profile of the soil bacteria. As already mentioned, CCA analysis revealed that pH explains 26% of total variance in the community profile (Supplementary Figure S3). Thus, the pH was the strongest predictor for bacterial community structure.

**Figure 4:**
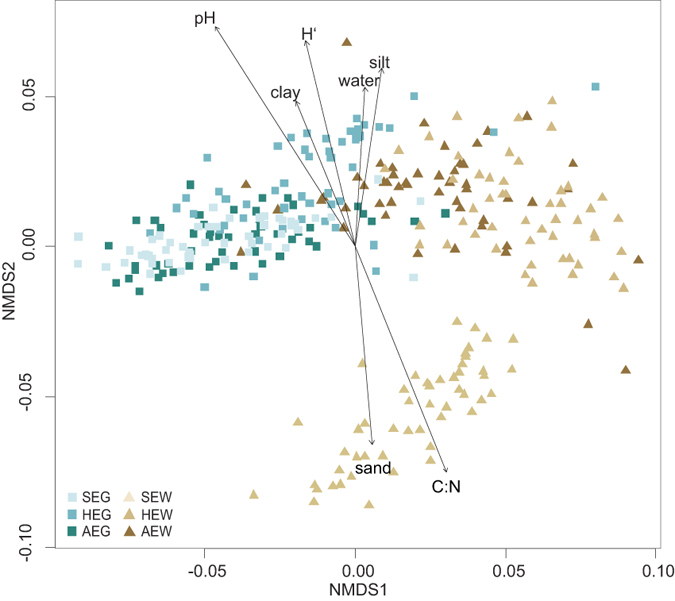
NMDS based on Bray Curtis dissimilarities of the functional profile. Statistically significant correlations of soil characteristics (C:N: carbon: nitrogen ratio; water: gravimetric water content; sand: sand amount; silt: silt amount; clay: clay amount) and the Shannon index (H’) were indicated by arrows. Grassland soil samples are represented by brown squares, forest samples by green triangles. Samples from different regions are distinguished by color shading (SEG: Schorfheide-Chorin grassland; SEW: Schorfheide-Chorin forest; HEG: Hainich-Dün grassland; HEW: Hainich-Dün forest; AEG: Schwäbische Alb grassland; AEW: Schwäbische Alb forest).

We hypothesized that bacterial community structure and functioning would be shaped in a similar manner. Environmental correlations with the Tax4Fun-derived functional profile were tested by NMDS based on Bray-Curtis dissimilarities (Fig. 4). The results are similar to those obtained for the community structure. The pH played an important role in shaping the functional profile and explained 32% of the variance (tested by CCA, P < 0.001, Supplementary Figure S5). This supports our hypothesis that structure and functions of bacterial communities are shaped by similar mechanisms. The functional profile also showed a separation between grassland and forest systems.

**Figure 5:**
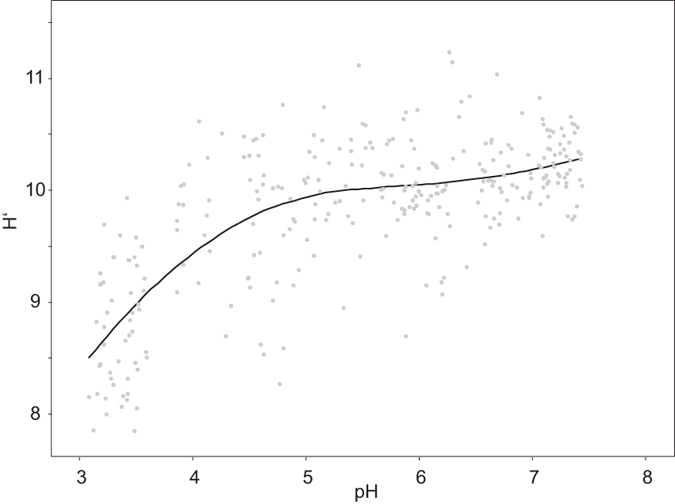
Relationship between soil bacterial diversity, represented by the Shannon index (H’) and soil pH. Points indicate observed Shannon indices for each sample, while the line represents the non-linear cubit regression fitted to the data (adjusted R^2^ =  0.5337, P < 0.001).

Additionally, we found that pH is the strongest predictor of soil bacterial diversity (P < 0.001, R^2^ = 0.4) (Fig. 5). It has already been shown that diversity of soil bacterial communities in the exploratories is positively correlated with pH^9^,^22^. However, our results indicate a more complex relationship between pH and diversity. Diversity was lowest at low pH, then increased and appeared to be stable between pH 5 and 7 and increases again under slightly alkaline conditions. This is in contrast to Fierer and Jackson^7^ and Lauber *et al.*^8^, who described a peak of soil bacterial diversity in near neutral soils.

### Multinomial regression models revealed multiple responses of bacterial orders to soil pH

**Figure 6:**
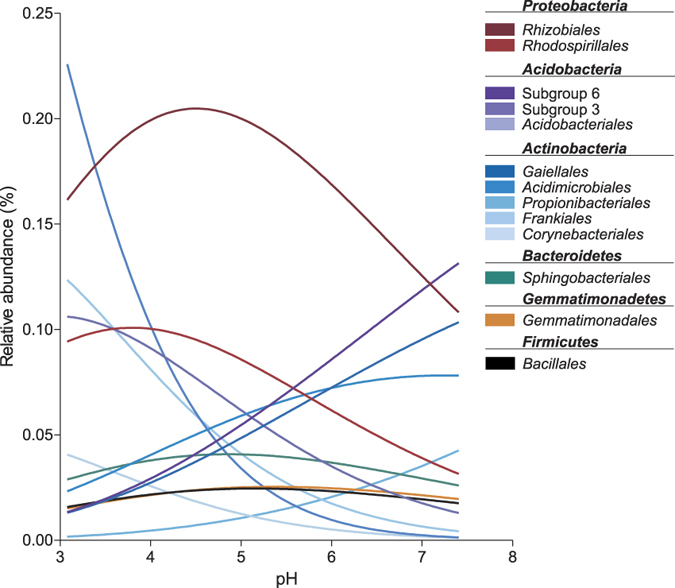
Response curves of selected bacterial orders towards pH. Each line represents the predicted abundance changes along the measured pH gradient, based on predictions derived from multinomial regression models. A detailed version of this graph including the 30 most abundant orders is available as Supplementary Material Figure S4.

To better understand the complex relationship of single bacterial groups and soil pH, we applied multinomial regression models on the 30 most abundant orders of the dataset (Supplementary Material Figure S6). Four general responses were observed: (1) decrease in abundance with increasing pH (*Acidobacteriales*, acidobacterial subgroup 3, *Frankiales, Corynebacteriales*), (2) increase in abundance with increasing pH (acidobacterial subgroup 6, *Gaiellales*, *Acidimicrobiales, Propionibacteriales*), (3) narrow pH range with high abundance (*Rhizobiales*, *Rhodospirillales*), and (4) relatively constant abundance across pH range (*Bacillales*, *Gemmatimonadales, Sphingobacteriales*) (Fig. 6). In their publication on niche theory, Austin and Smith^37^ described pH as a direct physiological gradient acting on organisms, resulting in unimodal, or skewed unimodal response curves restricted by growth limiting conditions at one end, and competition at the other end. This is supported by our observation of few highly abundant orders at low pH and many less abundant orders in near neutral soils. The ability to grow at low pH values is known as ATR (acid tolerance response) and confers a competitive advantage compared to other bacteria in soils.

**Figure 7:**
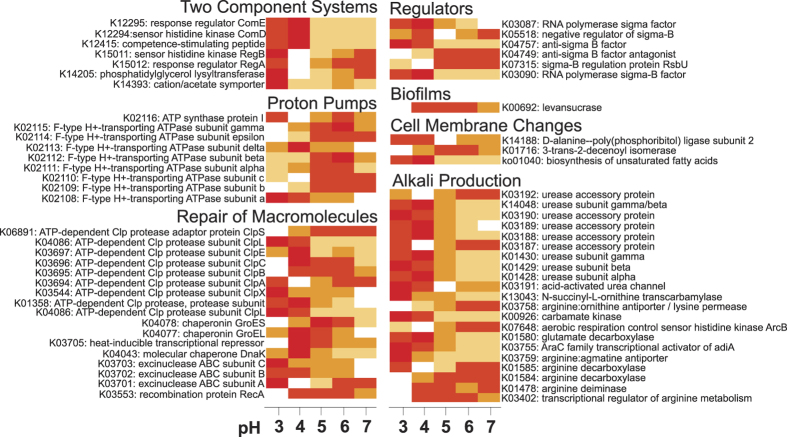
Heatmap based on mean abundances of genes putatively involved in ATR. Only genes with KEGG orthologs and present in the functional profile are shown. The KEGG pathway for biosynthesis of unsaturated fatty acids is included, also on the basis of the genes with KEGG orthologs in the functional profile. White: low relative abundance; yellow: mean relative abundance; red: high relative abundance.

To test which mechanisms are involved in acid tolerance of soil bacteria, we chose those genes reported to be involved in acid tolerance in *Rhizobia*^38^ and Gram positive bacteria^39^ that were present in the functional profile. Additionally, we analyzed the genes present of the KEGG pathway for biosynthesis of unsaturated fatty acids (ko01040) as well as 3-trans-2-decenoyl isomerase. This enzyme is involved in the generation of unsaturated fatty acids and was shown to increase acid tolerance in *Streptococcus mutans* by changing cell membrane composition^40^. We found that the genes for biosynthesis of unsaturated fatty acids were highly abundant in low pH samples (pH 3–4), while decenoyl isomerase did not follow this trend (Fig. 7). Therefore, this gene might not be generally involved in acid tolerance in soil. Additionally, most genes involved in alkali production, two component systems and repair of macromolecules were more abundant in the low pH samples compared to more neutral samples. Several genes involved in DNA repair were probably also involved in the ATR of soil-inhabiting bacteria, as well as levansucrase, a gene involved in biofilm formation. Our results suggest that bacteria can apply an active mechanism to cope with stressful pH conditions. Alkali production increases the pH in the immediate environment, improving bacterial survival chances. Additionally, macromolecule repair-enzymes protect and repair DNA and proteins, and bacteria seem to enhance pH tolerance by altering their cell wall components or protect themselves within biofilms.

## Conclusion

During the last years, several studies targeting soil microbial communities and their driving forces came to the same conclusion that soil pH is the major driver of bacterial communities. This statement, however, falls short as it provides no direct answer about the complex interaction of soil bacteria with pH. We showed that soil bacteria respond differently to changing pH conditions, being adapted to certain pH ranges or even stable over a broad pH range. Obtained data suggest that this adaptation is attributed to different mechanisms including alkali production and alteration of cell wall components. In addition to soil pH, it is generally assumed that land use intensity drives bacterial community composition and diversity. However, the present study demonstrated that land use intensity plays a minor role, or that its effect is concealed by the tree species effect in forest. Biogeographic variations and the corresponding changing edaphic properties resulted in distinct patterns of soil bacteria, which explains regional differences and also the distinct patterns of bacterial communities in grasslands and forests. This is in line with our first and second hypothesis.

Large comparative studies are required to unravel the diverse interactions between bacteria and their environments, and how changes in community structure might reflect changes in bacterial functioning. With a total of 300 samples representing different land uses and gradients of land use intensity, this study provides comprehensive insights into soil bacterial communities present in temperate systems. Taking the enormous size and diversity of soil microbial communities into account, functional information on soil bacterial communities has been limited as it was so far mainly derived from small-scale comparative metagenomic approaches with a rather low coverage. However, the ability to focus on functional genes and enzymes offers novel insights in the nutrient cycling potential of soil bacterial communities. Consequently, the application of novel bioinformatic and statistical approaches, such as Tax4Fun and multinomial log-linear models, in microbial ecology resulted in a more holistic understanding of the links between bacteria and their environment.

## Materials and Methods

### Study regions

The present study was conducted as part of the German Biodiversity Exploratories initiative, which is a project investigating large-scale and long-term relationships of biodiversity and land use in Central European grasslands and forests^21^. Its unique design allows detailed analysis of bacterial communities along a regional north-south gradient in Germany. The study is based on 300 plots in three study regions (exploratories). They are located in the Schorfheide-Chorin, the Hainich-Dün and the Schwäbische Alb. Each study region covers the land use types forest and grassland. Grassland plots are 50 m × 50 m and forest plots are 100 m × 100 m in size.

The grassland land use intensity-gradient was represented by three different management regimes (meadows, pastures and mown pastures) that are non-fertilized or fertilized. Fertilization always represents higher land use intensity. The land use intensity index (LUI^41^) combines and equally weights the three components of land use in grasslands: (1) fertilization, (2) mowing, and (3) grazing. To account for interannual variation in management practices, the LUI was calculated from 2006 (start of the experiment) to 2011 (sampling year) (Supplementary Table S1). It is therefore used as an index for long-term management and thereby allows the evaluation of long-term effects on bacterial communities.

In forests, the land use intensity-gradient was represented by different forest management systems (age class forest, selection forest and unmanaged forest). Additionally, forest plots were dominated by one of the following tree species: (1) European beech (*Fagus sylvatica*), (2) sessile/pedunculate oak (*Quercus petrea*/*Quercus robur*), (3) Scots pine (*Pinus sylvestris*) or (4) Norway spruce (*Picea abies*). The silvicultural management index (SMI) was used to assess the impact of management intensity in forest systems (Supplementary Table S1). This index integrates three characteristics of forest stands: (1) tree species, (2) stand age and (3) aboveground, living and dead wooden biomass^42^. Detailed information on land use, the applied management, dominant tree species, soil type and fertilization for every experimental plot is provided in Supplementary Material Table S2.

### Sampling and soil properties

Soil samples were collected from all 300 experimental plots in May 2011. In brief, plots were sampled along two 36 m transects in forests and along two 18 m transects in grasslands. The top 10 cm of the soil layer were taken from 14 locations along the two transects in each plot with a split tube auger of 5 cm diameter. At forest sites, the litter layer was removed with a metal frame (15 × 15 cm) prior to sampling. The soil cores were pooled and sieved to remove stones >0.5 cm and roots.

Ten grams of the pooled soil samples were used to determine the gravimetric water content, which represents the water content of the respective sample at the sampling time. The subsamples were weighted and dried at 105 °C to a constant weight. Air-dried soil samples sieved to <2 mm were used for the determination of soil texture, soil pH, and carbon (C) and nitrogen (N) concentrations as described previously^43^. Detailed information on soil characteristics is given in Supplementary Material Table S1.

### DNA extraction, amplification of 16S rRNA genes and pyrosequencing

Total microbial community DNA was isolated from approximately 0.25 g soil per sample using the MoBio Power Soil DNA isolation kit (MoBio laboratories, Carlsbad, CA, USA) following the manufacturer’s recommendations. This method was recently shown to perform equally well over a range of different soils^44^. It produces similar amounts of DNA and 16S rRNA gene copies for each soil tested and does not overestimate any of the abundant phyla detected throughout the soils. Therefore, extraction biases were limited and comparability given for all DNA extractions. DNA concentrations were quantified using a NanoDrop ND-1000 UV-Vis Spectrophotometer (NanoDrop Technologies, USA) as recommended by the manufacturer.

The V3-V5 region of the 16S rRNA gene was amplified by PCR. The PCR reaction mixture (50 μl) contained 10 μl 5-fold reaction buffer, 200 μM of each of the four deoxyribonucleoside triphosphates, 2% DMSO, 2% BSA, 0.2 μM of each of the primers, 0.5 U of Phusion High fidelity DNA polymerase (Thermo Scientific, Waltham, MA, USA) and approximately 50 ng of isolated DNA as template. The V3-V5 region was amplified with the following set of primers containing the Roche 454 pyrosequencing adaptors and a unique MID per sample (underlined): V3for 5′-CCATCTCATCCCTGCGTGTCTCCGACTCAG-MID-TACGGRAGGCAGCAG-3′^45^ and V5rev 5′-CCTATCCCCTGTGTGCCTTGGCAGTCTCAG-MID-CCGTCAATTCMTTTGAGT-3′^46^. The following thermal cycling scheme was used: initial denaturation at 98 °C for 3 min, 25 cycles of denaturation at 98 °C for 10 s, annealing at 58 °C for 30 s, and extension at 72 °C for 30 s followed by a final extension at 72 °C for 10 min. All samples were amplified in triplicate, pooled in equal amounts and purified by gel electrophoresis using peqGOLD Gel Extraction kit as recommended by the manufacturer (Peqlab Biotechnologie GmbH, Erlangen, Germany). PCR products were quantified using the Quant-iT dsDNA HS assay kit and a Qubit fluorometer (Invitrogen GmbH, Karlsruhe, Germany) as recommended by the manufacturer. The Göttingen Genomics Laboratory determined the 16S rRNA gene sequences employing the Roche GS-FLX+ pyrosequencer with Titanium chemistry (Roche, Mannheim, Germany).

### Analysis of pyrosequencing data

Pyrosequencing-derived 16S rRNA gene sequences were processed using the QIIME software package version 1.8^47^. Following the extraction of raw data, reads shorter than 300 bp, with long homopolymer stretches (>8 bp), or primer mismatches (>3) were removed. Subsequently, sequences were denoised employing Acacia version 1.53b^48^. Cutadapt^49^ was employed to truncate remaining primer sequences. Chimeric sequences were removed using UCHIME implemented in USEARCH version (8.0.1623) first in de novo and subsequently in reference mode using the SILVA SSURef 123 NR database as reference database^50^,^51^. Afterwards, processed sequences were clustered with UCLUST version 1.2.22q in operational taxonomic units (OTUs) at 97% and 80% genetic identity representing species and phylum level, respectively^52^. OTUs were classified by BLAST alignment against the most recent SILVA database (see above). Rarefaction curves, alpha diversity indices (Chao1, Shannon, Simpson) and Michaelis-Menten-Fit were determined using QIIME according to Wemheuer *et al.*^53^. The analysis was performed by using 5,311 sequences per sample (Supplementary Material Table S3). Non-metric multidimensional scaling plots were generated based on Bray Curtis dissimilarities or weighed UniFrac distances in R using the metaMDS function to visualize differences in bacterial community composition.

### Statistical analyses

All statistical analyses were conducted employing R version 3.1^54^. The results of all statistical tests were regarded significant with P ≤ 0.05, and only significant results are shown and described throughout the manuscript. The median is used throughout the manuscript instead of the mean value, except stated otherwise. For all statistical analysis, the dataset calculated for 97% identity (species level) was used.

The Mann-Whitney-test and non-parametric Kruskal-Wallis one-way analysis of variance (ANOVA) were used due to the non-normal distribution of the data. They were performed to test for differences in soil parameters and bacterial diversity between land use systems, exploratories and management regimes. The effects of environmental parameters onto the variance of bacterial communities were analyzed using the *envfit* function as described previously^55^. Canonical correspondence analysis (CCA) on single soil properties was carried out using the *cca* function and subsequently tested for significance applying the *permu.test* function with 1000 permutations. All these functions are contained in the vegan package^56^. Response curves of bacterial orders toward pH were calculated employing a multinomial log-linear model (function *multinom* contained in the nnet package).

Functional profiles were predicted from obtained 16S rRNA gene data using Tax4Fun^23^. Genes involved in acid tolerance (ATR) and encoding key enzymes in nutrient cycling were identified in the resulting profiles using their KEGG orthologs. The heatmap, based on the ATR-involved genes was calculated using the *heatmap.2* function of the gplots package^57^. Differences in the abundances of key genes involved in nutrient cycling were analyzed employing the Mann-Whitney test in R. The mean abundances of genes in grasslands and forests (relative to mean abundance in complete dataset) were plotted against each other using *ggplot* of the ggplot2 package^58^.

### Sequence data deposition

Sequence data were deposited in the Sequence Read Archive (SRA) of the National Center for Biotechnology Information (NCBI) under the accession number SRP065604.

## Additional Information

**How to cite this article**: Kaiser, K. *et al.* Driving forces of soil bacterial community structure, diversity, and function in temperate grasslands and forests. *Sci. Rep.* **6**, 33696; doi: 10.1038/srep33696 (2016).

## Supplementary Material

Supplementary Information

## Figures and Tables

**Table 1:** Edaphic properties among different land uses and exploratories (median ± SD).

Land use	Exploratory	n	pH	C:N ratio	Gravimetric water content (%)	Clay (g kg^−1^)	Silt (g kg^−1^)	Sand (g kg^−1^)
Forest	All plots	150	4.5 ± 1.1^A^	13.8 ± 3.2^A^	33.5 ± 18.1	289.0 ± 203.3^A^	440.5 ± 247.1	67.5 ± 386.7
	Schorfheide-Chorin	50	3.4 ±0.1^a^	18.1 ± 2.8^a^	12.0 ± 4.3^a^	48.5 ± 18.9^a^	74.0 ± 49.2^a^	875.0 ± 60.6^a^
	Hainich-Dün	50	4.6 ± 0.9^b^	12.8 ± 1.1^b^	33.5 ± 6.4^b^	307.0 ± 99.3^b^	634.5 ± 95.6^b^	54.5 ± 17.5^b^
	Schwäbische Alb	50	5.2 ± 0.8^b^	12.9 ± 0.9^b^	52.5 ± 10.0^c^	501.0 ± 104.8^c^	445.0 ± 107.6^c^	42.5 ± 46.0^b^
Grassland	All plots	150	6.7 ± 0.7^B^	10.3 ± 0.9^B^	31.5 ± 39.4	425.0 ± 192.4^B^	418.0 ± 159.3	74.5 ± 228.2
	Schorfheide-Chorin	50	6.4 ± 0.9^a^	10.4 ± 1.1^a^	54.5 ± 60.5^a^	159.5 ± 87.0^a^	317.0 ± 191.7^a^	489.5 ± 220.8^a^
	Hainich-Dün	50	7.1 ± 0.9^b^	10.1 ± 0.5^b^	22.0 ± 5.4^b^	452.0 ± 130.3^b^	489.5 ± 122.7^b^	53.5 ± 23.1^b^
	Schwäbische Alb	50	6.2 ± 0.5^a^	10.2 ± 0.7^b^	41.0 ± 11.1^a^	571.0 ± 134.0^c^	386.0 114.6^a^	41.0 ± 45.0^b^

Significant differences between study regions are indicated by lowercase letters and between forest and grassland by capital letters according to Dunn’s test (P < 0.05).
