# Supplementary material for: Driving forces of soil bacterial community structure, diversity, and function in temperate grasslands and forests
Source: Sci Rep. 2016 Sep 21;6:33696. doi: 10.1038/srep33696 (PMC5030646; doi:10.1038/srep33696)
Supplement: Supplementary Information [file srep33696-s1.pdf]

Supplementary Material

Driving forces of soil bacterial community structure, diversity, and function in temperate grasslands and forests

Kristin Kaiser, Bernd Wemheuer, Vera Korolkow, Franziska Wemheuer, Heiko Nacke, Ingo Schöning, Marion Schrumpf and Rolf Daniel

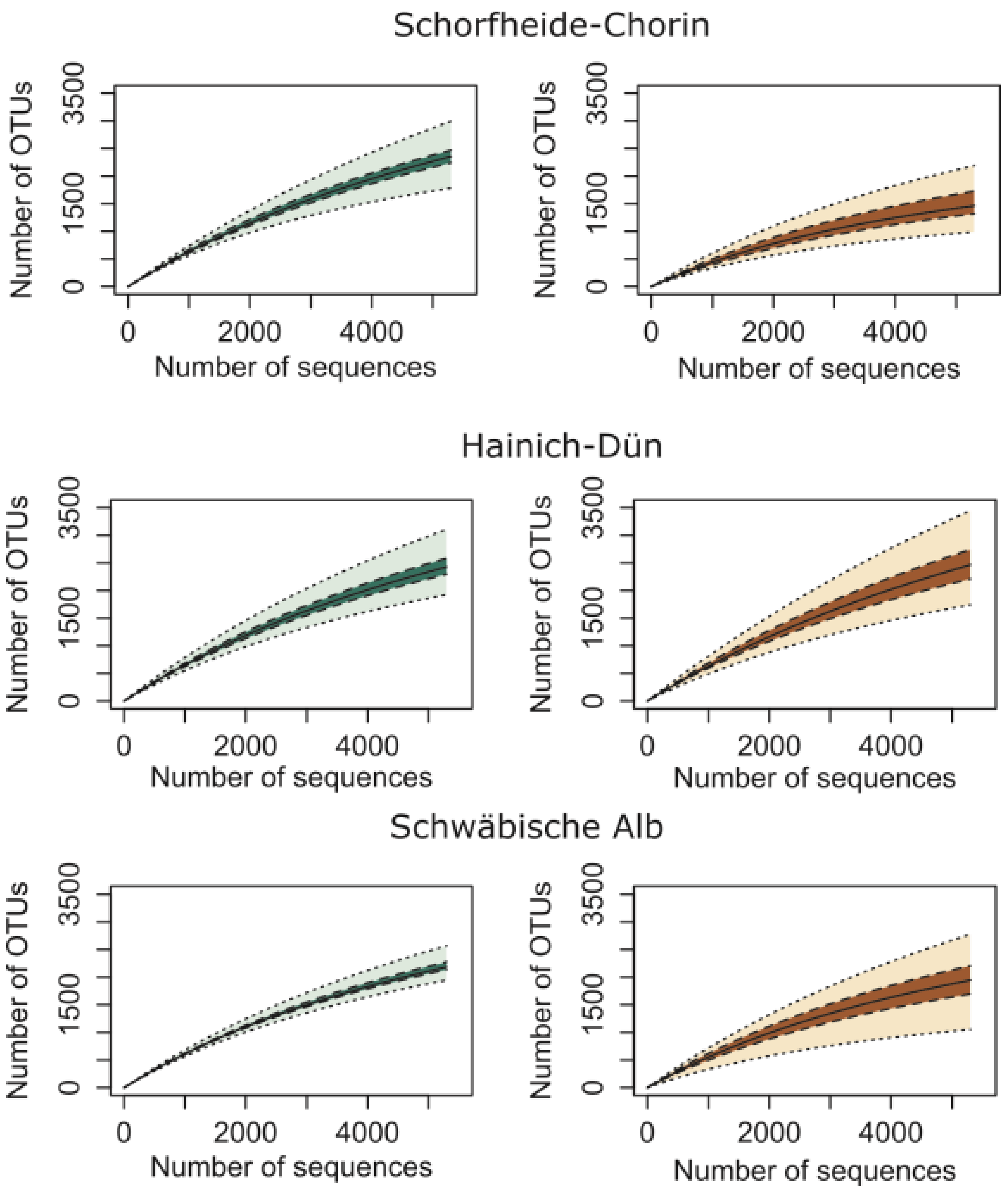

Fig. S1. Rarefaction curves at 3% genetic distance. Rarefaction was calculated for a subset of 5,311 sequences per sample to prevent artificial overestimation of OTUs based on higher sequence numbers. Green rarefaction curves represent grassland samples, brown rarefaction curves, represent forest samples.

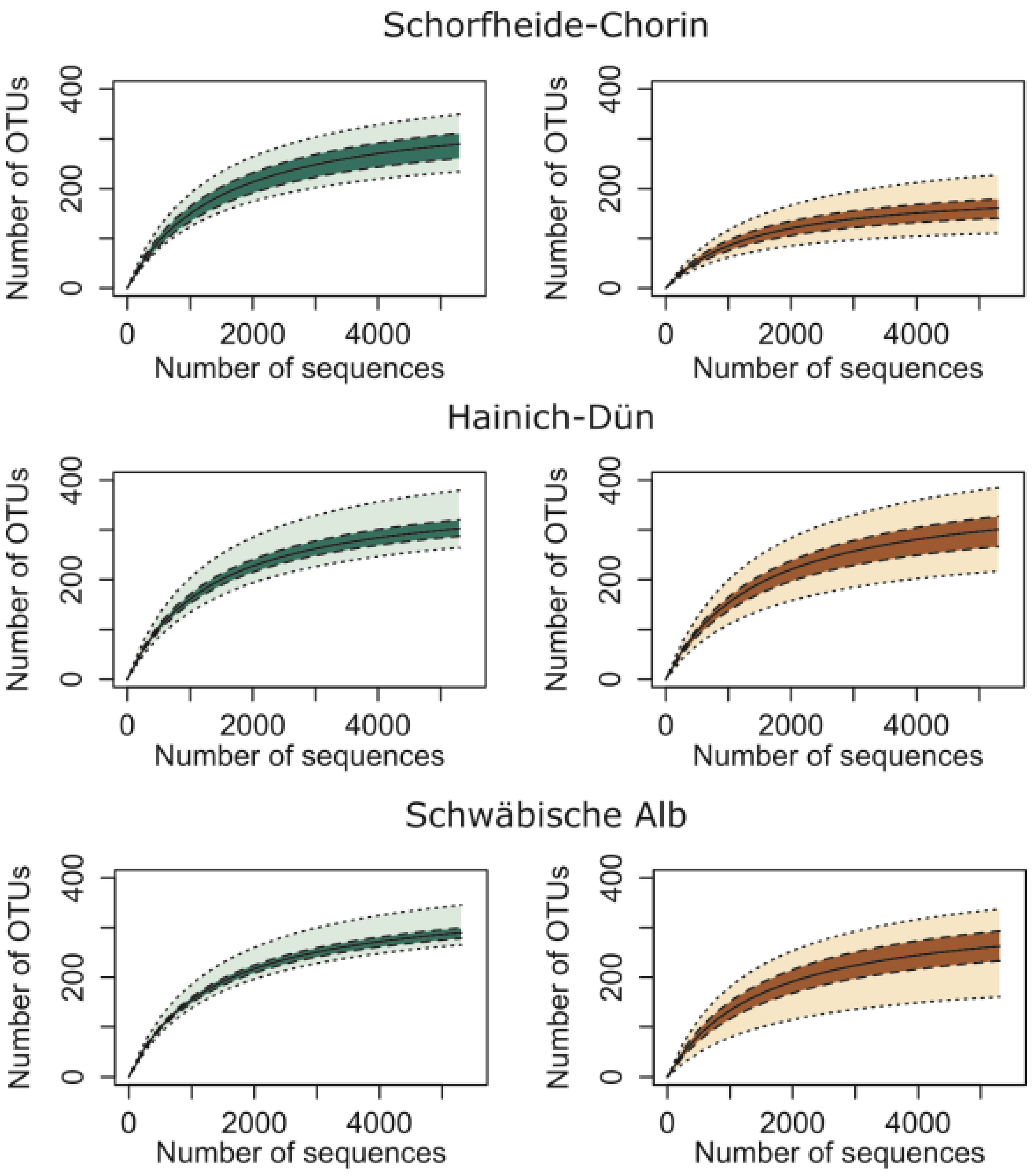

Fig. S2. Rarefaction curves at 20% genetic distance. Rarefaction was calculated for a subset of 5,311 sequences per sample to prevent artificial overestimation of OTUs based on higher sequence numbers. Green rarefaction curves represent grassland samples, brown rarefaction curves, represent forest samples.

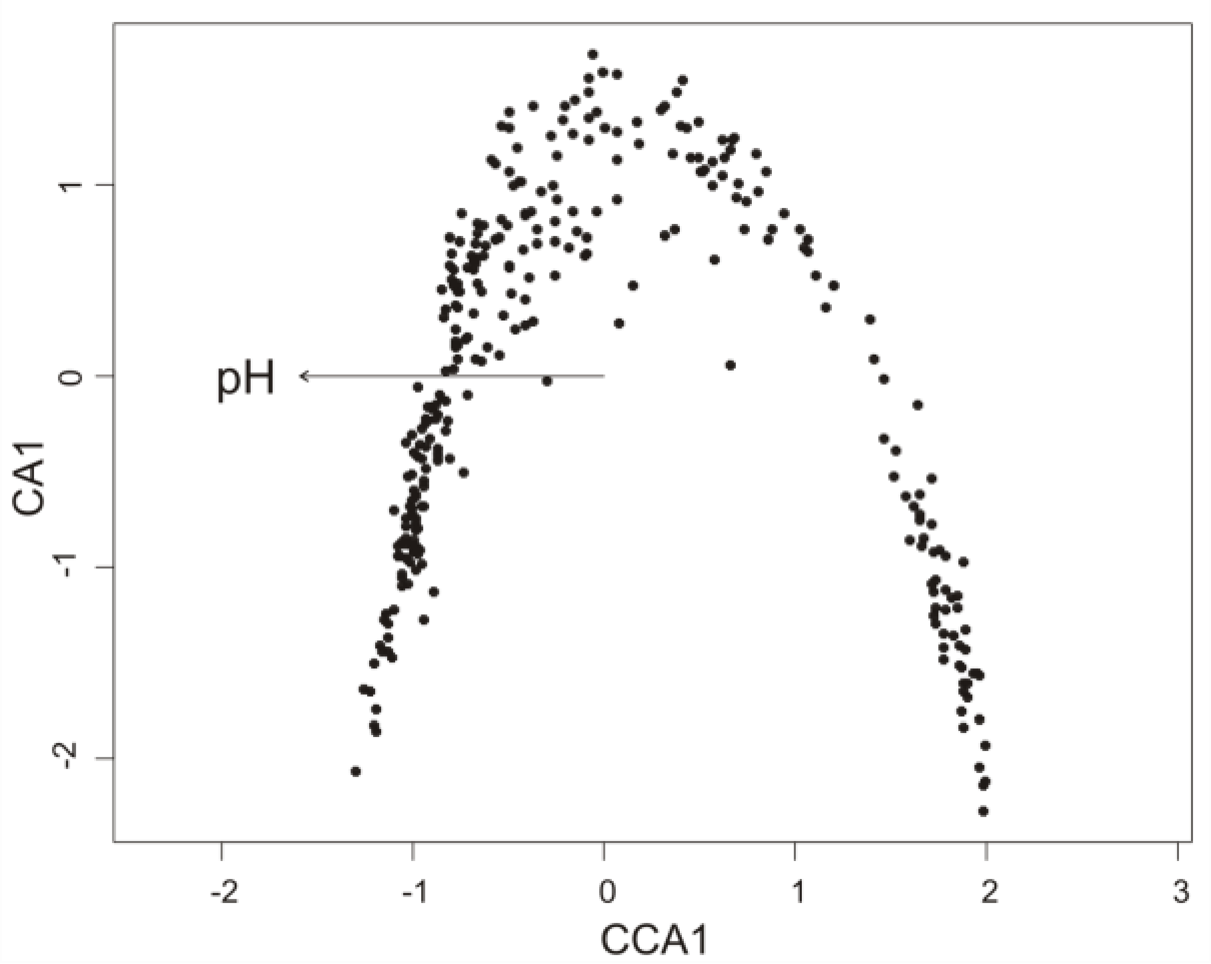

Fig. S3. Canonical correspondence analysis of the community profile using pH as constrained variable.

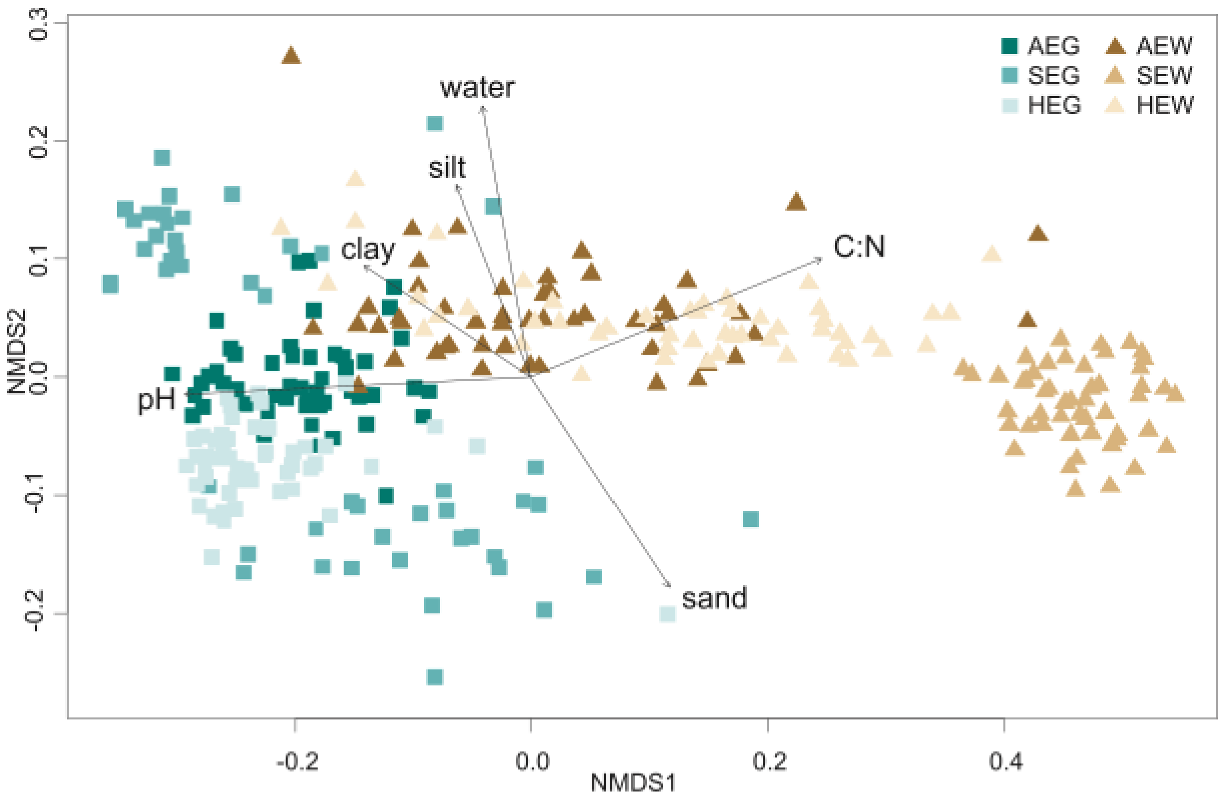

Fig. S4. NMDS based on Bray Curtis dissimilarities of the taxonomic profile. Statistically significant correlations of soil characteristics (C:N: carbon : nitrogen ratio; water: gravimetric water content; sand: sand amount; silt: silt amount; clay: clay amount) were indicated by arrows. Green squares represent forest samples (AEG: Schwäbische Alb grassland; SEG: Schorfheide-Chorin grassland; HEG: Hanich-Dün grassland), brown triangles represent forest samples (AEW: Schwäbische Alb forest; SEW: Schorfheide-Chorin forest; HEW: Hainich-Dün forest).

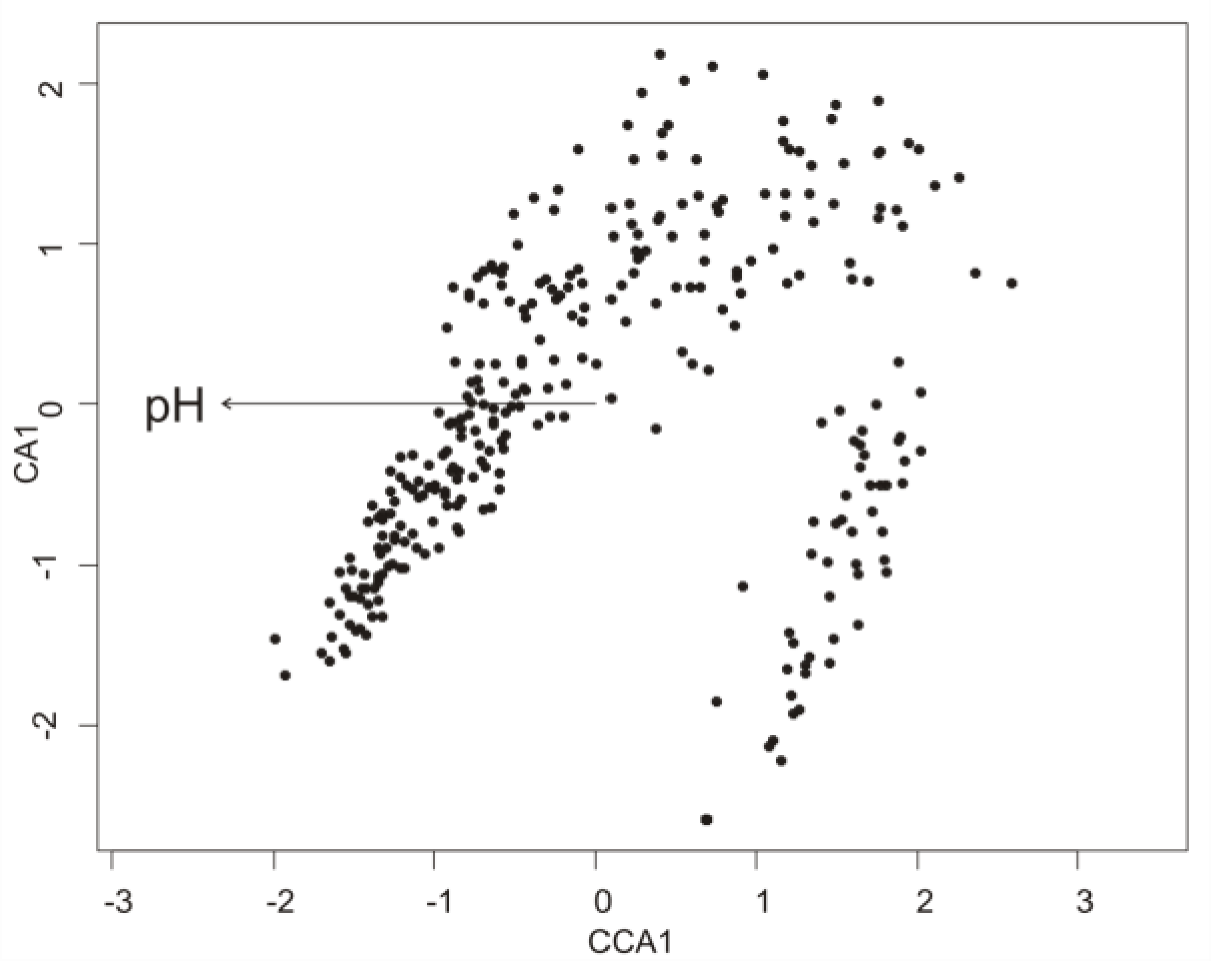

Fig. S5. Canonical correspondence analysis of the functional profile using pH as constrained variable.

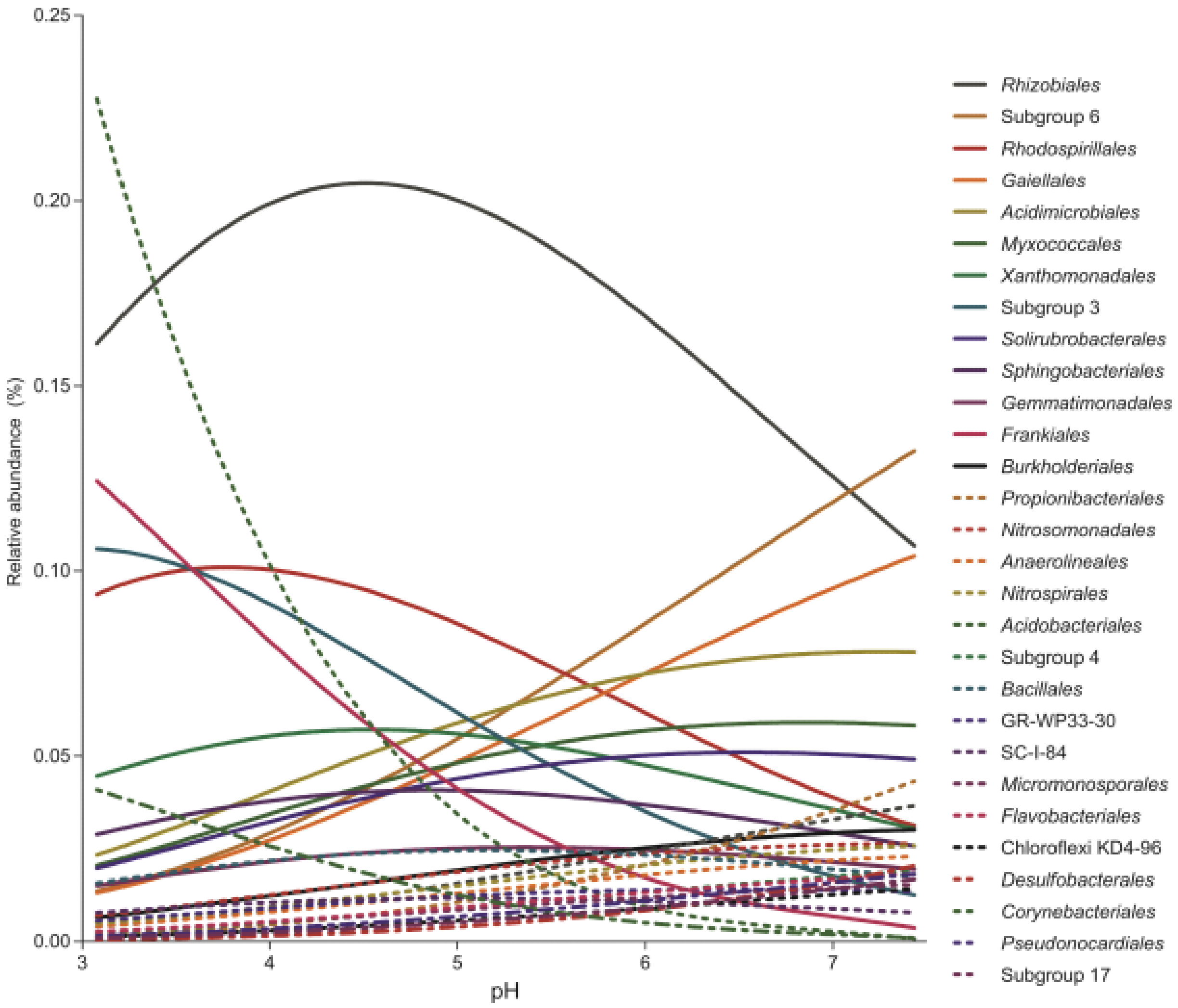

Fig. S6: Response curves of the 30 most abundant bacterial orders (representing >1% of the total community) toward pH. Each line represents the predicted abundance changes along the measured pH gradient, based on predictions derived from multinomial regression models.

Table S1. Edaphic parameters. This table gives the pH, C:N ratio (C:N), gravimetric water content (water) and amounts of clay, silt and sand; LUI: land use intensity index, calculated for 2006-2011, as described in Blüthgen et al. (2012) for grassland samples; SMI: silvicultural management index (Schall & Ammer, 2013) for forest samples.

**Site**	**pH**	**C:N**	**Water (%)**	**Clay (g kg-1)**	**Silt (g kg-1)**	**Sand (g kg-1)**	**LUI / SMI**
SEG1	7.22	10.65	134	233	466	301	2.33
SEG2	7.32	10.1	90	198	527	275	2.14
SEG3	7.42	10.1	84	89	600	311	2.28
SEG4	7.35	10.15	113	163	686	151	1.04
SEG5	7.41	10	88	162	575	263	1.08
SEG6	5.5	12.46	165	248	284	468	1.48
SEG7	7.29	10.42	57	157	648	195	1.64
SEG8	7.34	10.71	63	110	713	177	1.06
SEG9	6.56	12.6	114	171	143	686	1.53
SEG10	7.39	10.36	127	183	516	301	2.19
SEG11	7.42	10	101	137	552	311	2.19
SEG12	7.37	10	98	138	548	314	2.33
SEG13	7.12	10	10	41	324	635	1.79
SEG14	7.34	10	107	169	625	206	1.04
SEG15	7.36	10	106	191	593	216	1.16
SEG16	7.4	10.07	88	178	655	167	1.15
SEG17	5.03	12.66	227	374	348	278	1.28
SEG18	5.14	12.3	4	65	89	846	1.36
SEG19	7.33	10.09	71	87	553	360	1.03
SEG20	6.58	12.51	133	307	304	389	1.37
SEG21	5.35	12.83	52	278	310	412	1.51
SEG22	7.28	10	51	93	636	271	1.62
SEG23	5.2	14.14	83	373	461	166	1.62
SEG24	7.07	10.08	149	283	504	213	1.16
SEG25	6.29	12.19	114	176	206	618	1.42
SEG26	7.14	12.29	161	427	353	220	1.51
SEG27	5.76	14.56	193	416	417	167	1.00
SEG28	7.29	10.66	107	175	651	174	1.16
SEG29	7.39	10.16	131	195	694	110	0.92
SEG30	7.09	10.52	10	146	343	511	1.29
SEG31	6.23	10.77	10	182	281	537	1.29
SEG32	5.91	10.5	8	190	234	576	1.29
SEG33	5.71	10.41	14	118	265	617	1.84
SEG34	5.58	10.59	8	138	273	589	1.94
SEG35	5.96	10.62	8	200	238	562	1.97
SEG36	6.2	10.09	13	139	301	560	1.56
SEG37	4.57	10.51	4	93	99	808	1.81
SEG38	5.17	10.46	4	89	72	838	2.44

Table S1 continued.

Site	pH	C:N	Water (%)	Clay (g kg-1)	Silt (g kg-1)	Sand (g kg-1)	LUI[a] / SMI[b]
SEG39	7.06	10.34	7	225	237	538	1.28
SEG40	5.75	10.49	6	98	192	710	2.11
SEG41	5.29	10.41	7	136	185	679	2.06
SEG42	5.08	10.06	6	117	289	594	2.74
SEG43	5.93	10.12	6	131	121	748	2.25
SEG44	5.41	10.15	6	113	189	698	1.37
SEG45	5.25	10.21	7	100	234	666	1.24
SEG46	5.36	10.58	12	146	210	644	1.77
SEG47	6.16	10.55	10	186	255	559	1.49
SEG48	6.73	10.11	4	130	120	750	1.59
SEG49	5.98	10.66	8	106	372	522	1.74
SEG50	5.07	10.84	6	92	112	796	1.58
SEW1	3.5	23.2	9	37	81	882	0.351
SEW2	3.4	17.07	11	32	98	870	0.329
SEW3	3.34	19.84	7	24	50	926	0.334
SEW4	3.27	20.75	11	27	54	919	0.136
SEW5	3.18	19.37	14	19	17	964	0.211
SEW6	3.45	17.39	15	49	77	874	0.319
SEW7	3.48	16.29	16	16	124	860	0.082
SEW8	3.28	16.54	23	47	198	755	0.059
SEW9	3.26	19.18	14	0	58	942	0.017
SEW10	3.58	17.94	7	36	53	911	0.381
SEW11	3.29	23.83	8	31	85	884	0.281
SEW12	3.38	26.3	7	18	106	876	0.391
SEW13	3.15	20.53	9	28	51	921	0.224
SEW14	3.17	17.63	10	8	17	975	0.276
SEW15	3.46	19.57	5	31	46	923	0.33
SEW16	3.41	18.28	8	30	108	862	0.335
SEW17	3.08	23.57	7	24	49	927	0.175
SEW18	3.42	21.97	10	29	34	941	0.262
SEW19	3.5	20.17	6	37	44	919	0.285
SEW20	3.59	23.59	7	42	70	892	0.357
SEW21	3.14	18.6	9	55	69	876	0.218
SEW22	3.36	17.86	13	34	71	895	0.178
SEW23	3.24	15.1	11	51	76	873	0.162
SEW24	3.44	15.8	15	46	142	812	0.048
SEW25	3.43	15.72	13	63	77	860	0.129
SEW26	3.51	15.18	12	52	66	886	0.051
SEW27	3.3	15.93	15	51	139	810	0.234
SEW28	3.35	14.76	13	51	141	808	0.121
SEW29	3.18	21.93	6	30	11	959	0.073
SEW30	3.12	23.06	15	50	172	778	0.266

Table S1 continued.

Site	pH	C:N	Water (%)	Clay (g kg-1)	Silt (g kg-1)	Sand (g kg-1)	LUI[a] / SMI[b]
SEW31	3.23	19.37	9	51	61	888	0.285
SEW32	3.21	19.78	13	51	141	808	0.282
SEW33	3.18	24.96	4	25	9	966	0.125
SEW34	3.22	22.22	10	42	69	889	0.217
SEW35	3.43	18.82	14	65	74	861	0.234
SEW36	3.21	17.26	16	64	88	848	0.166
SEW37	3.43	16.97	12	63	70	871	0.161
SEW38	3.18	15.96	16	57	103	840	0.204
SEW39	3.46	17.74	15	52	98	850	0.29
SEW40	3.56	17.73	13	63	71	866	0.25
SEW41	3.57	18.96	10	50	49	905	0.244
SEW42	3.55	16.85	22	74	247	679	0.224
SEW43	3.48	16.49	15	69	121	810	0.229
SEW44	3.52	18.47	10	75	69	856	0.21
SEW45	3.5	17.9	14	48	75	881	0.092
SEW46	3.3	15.87	17	63	132	805	0.088
SEW47	3.21	17.62	10	49	18	933	0.082
SEW48	3.49	16.46	21	85	190	725	0.07
SEW49	3.24	18.52	12	68	74	858	0.334
SEW50	3.41	16.08	18	79	99	822	0.116
HEG1	6.64	10	20	502	447	50	2.75
HEG2	7.18	10	23	552	396	52	2.62
HEG3	7.25	10	27	544	399	57	2.62
HEG4	6.65	10.42	26	511	414	75	2.09
HEG5	7.09	10	37	454	497	49	2.46
HEG6	5.95	10.33	15	257	698	45	1.96
HEG7	6.98	10	31	536	434	30	1.73
HEG8	7.15	10	22	494	452	56	1.44
HEG9	6.9	11.49	17	387	537	76	0.72
HEG10	6.39	10.9	29	436	532	30	1.48
HEG11	7.23	10.4	21	552	405	43	1.43
HEG12	6.98	10	24	497	448	55	2.83
HEG13	7.15	10	16	394	536	70	1.76
HEG14	6.27	10.75	18	450	491	60	2.25
HEG15	7.09	10.16	24	528	435	38	2.06
HEG16	6.59	10.02	26	555	405	39	1.03
HEG17	6.98	10.71	29	546	419	32	0.63
HEG18	7.31	11.74	20	457	446	97	0.68
HEG19	6.58	10.89	35	485	427	87	0.63
HEG20	5.4	11.66	13	239	661	102	0.77
HEG21	7.33	10.5	22	311	631	58	0.67
HEG22	6.91	10	14	446	467	87	1.35

Table S1 continued.

Site	pH	C:N	Water (%)	Clay (g kg-1)	Silt (g kg-1)	Sand (g kg-1)	LUI[a] / SMI[b]
HEG23	7.19	10	21	588	375	37	1.47
HEG24	6.63	9	26	545	404	52	1.61
HEG25	7.24	10	30	423	531	46	1.60
HEG26	7.29	10.1	24	481	458	61	1.30
HEG27	7.28	10.43	22	492	472	36	1.74
HEG28	7.22	10	17	536	409	55	1.96
HEG29	7.12	10.28	15	469	501	30	1.89
HEG30	7.1	10	14	400	548	52	2.28
HEG31	7.13	10.02	22	237	726	36	1.94
HEG32	5.52	10.54	21	340	640	17	2.02
HEG33	5.01	10.45	15	353	618	29	1.99
HEG34	7.06	10	16	448	469	83	1.96
HEG35	6.96	10	14	307	580	113	2.07
HEG36	7.24	10	22	61	871	71	2.15
HEG37	7.37	10	21	60	841	99	2.12
HEG38	7.28	10	16	310	622	68	1.64
HEG39	6.44	10	23	351	537	110	1.24
HEG40	6.68	10.08	22	275	648	79	1.55
HEG41	7.18	11.29	17	518	417	65	0.77
HEG42	7.15	10.91	28	435	520	46	0.63
HEG43	7.13	11.16	23	376	589	38	0.85
HEG44	7.13	10.32	22	79	853	69	0.73
HEG45	6.72	10.39	22	492	468	43	0.60
HEG46	7.43	10.4	19	395	531	74	0.77
HEG47	7.08	10	23	483	477	40	1.49
HEG48	6.78	10.33	19	465	488	50	1.39
HEG49	6.62	10	22	431	500	71	1.73
HEG50	6.81	10.27	20	646	335	23	1.81
HEW1	6.78	12.54	36	195	738	71	0.421
HEW2	5.52	15.23	23	308	600	92	0.387
HEW3	4.98	15.79	31	409	549	43	0.508
HEW4	5.99	12.66	45	387	562	55	0.463
HEW5	4.99	12.64	35	460	485	59	0.219
HEW6	4.15	12.46	30	218	709	74	0.101
HEW7	4.1	13.95	30	197	714	88	0.18
HEW8	5.56	13.93	35	227	716	58	0.195
HEW9	4.12	12.78	33	287	622	92	0.188
HEW10	4.86	12.5	46	447	503	53	0.055
HEW11	4.53	12.35	35	414	551	36	0.002
HEW12	4.05	13.41	29	168	713	117	0.069
HEW13	6.66	15.13	43	510	444	48	0.389
HEW14	4.53	13.21	40	448	504	47	0.445

Table S1 continued.

Site	pH	C:N	Water (%)	Clay (g kg-1)	Silt (g kg-1)	Sand (g kg-1)	LUI[a] / SMI[b]
HEW15	3.91	16.2	31	150	785	66	0.427
HEW16	4.74	12.1	33	322	628	53	0.333
HEW17	3.86	15.24	30	183	725	96	0.364
HEW18	4.78	12.99	38	272	684	41	0.205
HEW19	4.56	11.4	33	310	636	52	0.071
HEW20	5.97	12.97	33	253	708	40	0.174
HEW21	4.7	12.46	37	268	672	61	0.135
HEW22	4.62	13.94	30	184	747	68	0.125
HEW23	4.53	12.56	30	279	662	56	0.397
HEW24	4.05	13.73	32	162	793	44	0.195
HEW25	4.57	12.73	34	325	624	53	0.317
HEW26	4.45	14.95	35	150	796	54	0.184
HEW27	6.37	12.93	49	291	643	67	0.19
HEW28	6.29	13.8	50	306	634	60	0.27
HEW29	3.86	13.94	28	227	719	55	0.154
HEW30	3.85	12.57	30	358	595	48	0.187
HEW31	3.87	12.62	27	377	573	52	0.173
HEW32	3.92	12.84	32	345	593	64	0.2
HEW33	4.45	14.17	32	227	706	68	0.047
HEW34	4.62	11.84	35	380	571	52	0.069
HEW35	4.5	12.44	39	396	551	55	0.108
HEW36	4.55	12.84	36	356	558	84	0.13
HEW37	4.49	12.61	32	265	661	78	0.022
HEW38	5.15	13.04	47	511	440	54	0.051
HEW39	4.14	12.64	28	330	615	58	0.049
HEW40	5.46	12.05	39	406	563	33	0.007
HEW41	4.25	12.52	32	210	754	34	0.107
HEW42	3.96	14.34	30	184	760	60	0.121
HEW43	6.35	12.83	42	337	635	30	0.486
HEW44	6.26	13.42	42	373	586	43	0.486
HEW45	7.09	12.5	51	88	854	60	0.293
HEW46	3.91	12.7	35	387	570	45	0.115
HEW47	4.79	13.35	35	323	632	46	0.113
HEW48	4.13	13.21	27	273	687	44	0.147
HEW49	3.89	14.83	27	223	732	48	0.191
HEW50	4.75	11.91	38	349	606	46	0.184
AEG1	6.53	10	40	643	324	33	1.87
AEG2	6.75	9	41	512	411	77	3.14
AEG3	6.22	10.21	56	672	298	30	2.03
AEG4	5.07	9.17	32	488	427	85	1.78
AEG5	6.14	10.34	59	365	518	117	1.85
AEG6	5.87	10.07	44	588	380	32	2.26

Table S1 continued.

Site	pH	C:N	Water (%)	Clay (g kg-1)	Silt (g kg-1)	Sand (g kg-1)	LUI[a] / SMI[b]
AEG7	7.11	11.37	33	385	427	188	0.51
AEG8	6.51	11.21	39	673	319	8	1.30
AEG9	6.7	11.91	50	556	417	27	0.61
AEG10	6.07	10.29	72	647	327	26	1.00
AEG11	5.49	10.39	55	643	327	30	2.13
AEG12	6.56	10.25	36	436	512	52	2.16
AEG13	5.95	10.39	38	643	311	46	2.16
AEG14	6.71	10.09	35	521	453	26	2.25
AEG15	5.67	10	37	642	340	18	2.59
AEG16	6.6	10.11	45	528	449	23	1.71
AEG17	6.86	9.89	47	618	365	17	2.17
AEG18	6.87	10	44	649	332	19	2.42
AEG19	5.85	10.17	56	637	327	36	2.41
AEG20	6.62	9	71	597	334	69	1.66
AEG21	5.99	10	29	587	391	22	3.92
AEG22	5.88	11.16	60	708	270	22	1.30
AEG23	6.77	9.83	49	566	387	47	1.36
AEG24	6.08	10.31	48	638	329	33	2.22
AEG25	6.97	11.27	62	463	406	131	0.83
AEG26	6.66	12.12	34	679	241	80	1.43
AEG27	6.14	11.63	50	576	410	14	1.12
AEG28	6.07	11.19	39	599	385	16	0.70
AEG29	5.77	10.32	25	566	343	91	1.49
AEG30	6.64	10	33	556	375	69	1.52
AEG31	6.55	10.18	34	595	381	24	1.19
AEG32	5.3	10.64	46	461	466	73	0.59
AEG33	5.97	10.61	41	597	388	15	1.21
AEG34	6.17	10	37	423	554	23	1.03
AEG35	5.67	10	33	446	503	51	1.76
AEG36	6.01	10.38	39	588	213	199	1.83
AEG37	6.1	10	28	566	334	100	1.91
AEG38	5.5	10	36	451	468	81	1.54
AEG39	5.67	10.52	32	463	493	44	2.00
AEG40	6.71	10	52	698	269	33	2.04
AEG41	6.15	10.26	33	541	407	52	2.41
AEG42	6.97	9	48	159	757	84	2.21
AEG43	6.81	10	54	613	349	38	1.87
AEG44	7.05	10	37	152	735	113	1.81
AEG45	5.19	10	50	529	417	54	1.48
AEG46	5.68	10	41	659	287	54	1.55
AEG47	7.18	10.8	41	146	704	150	0.80
AEG48	7.3	12.84	21	295	565	140	0.65

Table S1 continued.

Site	pH	C:N	Water (%)	Clay (g kg-1)	Silt (g kg-1)	Sand (g kg-1)	LUI[a] / SMI[b]
AEG49	6.16	10.36	45	451	522	27	0.96
AEG50	5.93	10.15	54	589	382	29	2.30
AEW1	3.42	15.34	41	318	659	23	0.54
AEW2	4.34	13.91	39	500	462	38	0.568
AEW3	5.65	14.18	38	527	424	49	0.515
AEW4	6.19	12.75	67	487	482	32	0.229
AEW5	4.5	12.98	54	404	579	18	0.183
AEW6	4.88	12.32	50	415	494	91	0.246
AEW7	4.59	13.05	59	548	362	90	0.15
AEW8	6.06	14.11	51	492	466	42	0.011
AEW9	5.88	14.29	46	693	289	18	0.127
AEW10	4.62	13.48	39	528	441	33	0.476
AEW11	3.48	15.09	35	260	699	41	0.486
AEW12	4.29	13.16	51	570	405	25	0.602
AEW13	4.76	14.85	54	666	289	45	0.433
AEW14	4.59	12.81	36	449	508	43	0.472
AEW15	6.21	13.76	72	653	277	70	0.406
AEW16	6.2	13.81	58	521	335	144	0.348
AEW17	6.41	12.5	56	428	540	32	0.225
AEW18	4.61	12.71	47	313	655	34	0.207
AEW19	4.8	12.08	50	471	503	28	0.15
AEW20	6.64	12.14	72	437	533	30	0.165
AEW21	6.11	11.71	59	519	453	30	0.124
AEW22	6.25	13.31	65	542	409	49	0.175
AEW23	5.48	12.42	55	500	486	15	0.177
AEW24	4.88	13.04	44	510	307	183	0.321
AEW25	4.7	12.9	38	383	506	111	0.418
AEW26	4.85	12.19	52	480	329	181	0.318
AEW27	4.49	13.8	47	518	352	130	0.334
AEW28	4.9	12.75	54	474	474	52	0.211
AEW29	4.48	13.92	43	413	550	37	0.487
AEW30	5.93	12.55	75	700	263	37	0.212
AEW31	5.8	12.2	46	548	431	21	0.538
AEW32	6.58	12.95	42	395	548	57	0.674
AEW33	5.33	14.4	44	681	305	14	0.528
AEW34	4.93	13.7	38	626	353	23	0.518
AEW35	5.1	14.34	58	587	362	51	0.379
AEW36	5.84	12.06	59	407	541	52	0.421
AEW37	5.39	12.44	63	553	374	73	0.301
AEW38	6.14	13.42	54	502	387	111	0.34
AEW39	5.73	13.1	55	432	530	38	0.242
AEW40	5.01	11.46	59	610	347	44	0.119

Table S1 continued.

Site	pH	C:N	Water (%)	Clay (g kg-1)	Silt (g kg-1)	Sand (g kg-1)	LUI[a] / SMI[b]
AEW41	5.12	12.3	55	475	449	76	0.172
AEW42	5.88	12.57	63	504	386	110	0.241
AEW43	4.8	11.06	48	386	577	39	0.278
AEW44	5.86	12.31	57	429	528	43	0.292
AEW45	5.71	12.22	53	220	574	206	0.274
AEW46	5.06	12.85	57	546	425	27	0.253
AEW47	5.07	12.84	50	531	404	65	0.199
AEW48	5.4	12.18	47	464	508	28	0.156
AEW49	5.85	12.65	47	645	234	121	0.238
AEW50	5.86	13.19	73	543	429	28	0.134

Table S2. Categorical Parameters. This table lists the exploratory (ALB: Schwäbische Alb; SCH: Schorfheide-Chorin; HAI: Hainich-Dün), land use, management type (AKL: age class forest; NW: unmanaged forest; PLW: selection forest), treatment (fertilized/non-fertilized), dominant tree species and soil type for all plots analyzed.

Site	Exploratory	Land use	Management	Treatment	Tree species	Soil type
SEG1	SCH	grassland	meadow	fertilized	NA	Histosol
SEG2	SCH	grassland	meadow	fertilized	NA	Histosol
SEG3	SCH	grassland	meadow	fertilized	NA	Histosol
SEG4	SCH	grassland	mown pasture	non-fertilized	NA	Histosol
SEG5	SCH	grassland	mown pasture	non-fertilized	NA	Gleysol
SEG6	SCH	grassland	mown pasture	non-fertilized	NA	Histosol
SEG7	SCH	grassland	pasture	non-fertilized	NA	Histosol
SEG8	SCH	grassland	pasture	non-fertilized	NA	Gleysol
SEG9	SCH	grassland	pasture	non-fertilized	NA	Histosol
SEG10	SCH	grassland	meadow	fertilized	NA	Histosol
SEG11	SCH	grassland	meadow	fertilized	NA	Gleysol
SEG12	SCH	grassland	meadow	fertilized	NA	Histosol
SEG13	SCH	grassland	meadow	fertilized	NA	Cambisol
SEG14	SCH	grassland	pasture	non-fertilized	NA	Gleysol
SEG15	SCH	grassland	mown pasture	non-fertilized	NA	Histosol
SEG16	SCH	grassland	mown pasture	non-fertilized	NA	Gleysol
SEG17	SCH	grassland	mown pasture	non-fertilized	NA	Histosol
SEG18	SCH	grassland	meadow	non-fertilized	NA	Luvisol
SEG19	SCH	grassland	pasture	non-fertilized	NA	Gleysol
SEG20	SCH	grassland	pasture	non-fertilized	NA	Histosol
SEG21	SCH	grassland	pasture	non-fertilized	NA	Gleysol
SEG22	SCH	grassland	pasture	non-fertilized	NA	Gleysol
SEG23	SCH	grassland	meadow	non-fertilized	NA	Histosol
SEG24	SCH	grassland	meadow	non-fertilized	NA	Histosol
SEG25	SCH	grassland	meadow	non-fertilized	NA	Histosol
SEG26	SCH	grassland	meadow	non-fertilized	NA	Histosol
SEG27	SCH	grassland	meadow	non-fertilized	NA	Histosol
SEG28	SCH	grassland	meadow	non-fertilized	NA	Histosol
SEG29	SCH	grassland	meadow	non-fertilized	NA	Histosol
SEG30	SCH	grassland	meadow	non-fertilized	NA	Albeluvisol
SEG31	SCH	grassland	meadow	non-fertilized	NA	Cambisol
SEG32	SCH	grassland	meadow	non-fertilized	NA	Luvisol
SEG33	SCH	grassland	mown pasture	fertilized	NA	Albeluvisol
SEG34	SCH	grassland	mown pasture	fertilized	NA	Albeluvisol
SEG35	SCH	grassland	mown pasture	fertilized	NA	Luvisol
SEG36	SCH	grassland	pasture	non-fertilized	NA	Albeluvisol
SEG37	SCH	grassland	pasture	non-fertilized	NA	Albeluvisol
SEG38	SCH	grassland	mown pasture	non-fertilized	NA	Cambisol
SEG39	SCH	grassland	pasture	fertilized	NA	Cambisol
SEG40	SCH	grassland	pasture	fertilized	NA	Luvisol

Table S2 continued.

Site	Exploratory	Land use	Management	Treatment	Tree species	Soil type
SEG41	SCH	grassland	pasture	fertilized	NA	Luvisol
SEG42	SCH	grassland	pasture	non-fertilized	NA	Luvisol
SEG43	SCH	grassland	pasture	non-fertilized	NA	Luvisol
SEG44	SCH	grassland	pasture	fertilized	NA	Cambisol
SEG45	SCH	grassland	pasture	fertilized	NA	Albeluvisol
SEG46	SCH	grassland	pasture	non-fertilized	NA	Cambisol
SEG47	SCH	grassland	pasture	non-fertilized	NA	Luvisol
SEG48	SCH	grassland	pasture	non-fertilized	NA	Luvisol
SEG49	SCH	grassland	pasture	non-fertilized	NA	Albeluvisol
SEG50	SCH	grassland	pasture	non-fertilized	NA	Cambisol
SEW1	SCH	forest	AKL	NA	pine	Cambisol
SEW2	SCH	forest	AKL	NA	pine	Cambisol
SEW3	SCH	forest	AKL	NA	pine	Cambisol
SEW4	SCH	forest	AKL	NA	pine	Cambisol
SEW5	SCH	forest	AKL	NA	beech	Cambisol
SEW6	SCH	forest	AKL	NA	beech	Cambisol
SEW7	SCH	forest	NW	NA	beech	Cambisol
SEW8	SCH	forest	NW	NA	beech	Albeluvisol
SEW9	SCH	forest	NW	NA	beech	Cambisol
SEW10	SCH	forest	AKL	NA	pine	Cambisol
SEW11	SCH	forest	AKL	NA	pine	Cambisol
SEW12	SCH	forest	AKL	NA	pine	Cambisol
SEW13	SCH	forest	AKL	NA	pine	Podzol
SEW14	SCH	forest	AKL	NA	pine	Cambisol
SEW15	SCH	forest	AKL	NA	pine	Cambisol
SEW16	SCH	forest	AKL	NA	pine	Cambisol
SEW17	SCH	forest	AKL	NA	pine	Cambisol
SEW18	SCH	forest	AKL	NA	pine	Cambisol
SEW19	SCH	forest	AKL	NA	pine	Cambisol
SEW20	SCH	forest	AKL	NA	pine	Regosol
SEW21	SCH	forest	AKL	NA	pine	Cambisol
SEW22	SCH	forest	AKL	NA	oak	Cambisol
SEW23	SCH	forest	AKL	NA	oak	Cambisol
SEW24	SCH	forest	AKL	NA	oak	Cambisol
SEW25	SCH	forest	AKL	NA	oak	Cambisol
SEW26	SCH	forest	AKL	NA	oak	Cambisol
SEW27	SCH	forest	AKL	NA	oak	Cambisol
SEW28	SCH	forest	AKL	NA	oak	Cambisol
SEW29	SCH	forest	AKL	NA	pine	Cambisol
SEW30	SCH	forest	AKL	NA	pine	Cambisol
SEW31	SCH	forest	AKL	NA	pine	Cambisol
SEW32	SCH	forest	AKL	NA	pine	Cambisol
SEW33	SCH	forest	AKL	NA	pine	Cambisol

Table S2 continued.

Site	Exploratory	Land use	Management	Treatment	Tree species	Soil type
SEW34	SCH	forest	AKL	NA	pine	Albeluvisol
SEW35	SCH	forest	AKL	NA	beech	Cambisol
SEW36	SCH	forest	AKL	NA	beech	Cambisol
SEW37	SCH	forest	AKL	NA	beech	Cambisol
SEW38	SCH	forest	AKL	NA	beech	Cambisol
SEW39	SCH	forest	AKL	NA	beech	Cambisol
SEW40	SCH	forest	AKL	NA	beech	Cambisol
SEW41	SCH	forest	AKL	NA	beech	Cambisol
SEW42	SCH	forest	AKL	NA	beech	Regosol
SEW43	SCH	forest	AKL	NA	beech	Cambisol
SEW44	SCH	forest	AKL	NA	beech	Cambisol
SEW45	SCH	forest	AKL	NA	beech	Albeluvisol
SEW46	SCH	forest	NW	NA	beech	Albeluvisol
SEW47	SCH	forest	NW	NA	beech	Cambisol
SEW48	SCH	forest	AKL	NA	beech	Cambisol
SEW49	SCH	forest	AKL	NA	beech	Cambisol
SEW50	SCH	forest	AKL	NA	beech	Cambisol
HEG1	HAI	grassland	meadow	fertilized	NA	Cambisol
HEG2	HAI	grassland	meadow	fertilized	NA	Vertisol
HEG3	HAI	grassland	meadow	fertilized	NA	Vertisol
HEG4	HAI	grassland	mown pasture	fertilized	NA	Stagnosol
HEG5	HAI	grassland	mown pasture	fertilized	NA	Stagnosol
HEG6	HAI	grassland	mown pasture	fertilized	NA	Stagnosol
HEG7	HAI	grassland	pasture	non-fertilized	NA	Stagnosol
HEG8	HAI	grassland	pasture	non-fertilized	NA	Stagnosol
HEG9	HAI	grassland	pasture	non-fertilized	NA	Stagnosol
HEG10	HAI	grassland	meadow	fertilized	NA	Vertisol
HEG11	HAI	grassland	meadow	fertilized	NA	Stagnosol
HEG12	HAI	grassland	mown pasture	fertilized	NA	Stagnosol
HEG13	HAI	grassland	mown pasture	non-fertilized	NA	Stagnosol
HEG14	HAI	grassland	mown pasture	fertilized	NA	Stagnosol
HEG15	HAI	grassland	mown pasture	fertilized	NA	Stagnosol
HEG16	HAI	grassland	pasture	non-fertilized	NA	Stagnosol
HEG17	HAI	grassland	pasture	non-fertilized	NA	Stagnosol
HEG18	HAI	grassland	pasture	non-fertilized	NA	Vertisol
HEG19	HAI	grassland	pasture	non-fertilized	NA	Stagnosol
HEG20	HAI	grassland	pasture	non-fertilized	NA	Stagnosol
HEG21	HAI	grassland	pasture	non-fertilized	NA	Stagnosol
HEG22	HAI	grassland	mown pasture	fertilized	NA	Cambisol
HEG23	HAI	grassland	mown pasture	non-fertilized	NA	Stagnosol
HEG24	HAI	grassland	mown pasture	non-fertilized	NA	Stagnosol
HEG25	HAI	grassland	pasture	non-fertilized	NA	Cambisol
HEG26	HAI	grassland	meadow	fertilized	NA	Cambisol

Table S2 continued.

Site	Exploratory	Land use	Management	Treatment	Tree species	Soil type
HEG27	HAI	grassland	meadow	fertilized	NA	Cambisol
HEG28	HAI	grassland	mown pasture	fertilized	NA	Cambisol
HEG29	HAI	grassland	mown pasture	fertilized	NA	Cambisol
HEG30	HAI	grassland	mown pasture	fertilized	NA	Cambisol
HEG31	HAI	grassland	mown pasture	fertilized	NA	Cambisol
HEG32	HAI	grassland	mown pasture	fertilized	NA	Cambisol
HEG33	HAI	grassland	pasture	fertilized	NA	Cambisol
HEG34	HAI	grassland	mown pasture	fertilized	NA	Cambisol
HEG35	HAI	grassland	mown pasture	fertilized	NA	Cambisol
HEG36	HAI	grassland	mown pasture	fertilized	NA	Cambisol
HEG37	HAI	grassland	mown pasture	fertilized	NA	Cambisol
HEG38	HAI	grassland	pasture	non-fertilized	NA	Cambisol
HEG39	HAI	grassland	pasture	non-fertilized	NA	Cambisol
HEG40	HAI	grassland	pasture	non-fertilized	NA	Cambisol
HEG41	HAI	grassland	pasture	non-fertilized	NA	Cambisol
HEG42	HAI	grassland	pasture	non-fertilized	NA	Cambisol
HEG43	HAI	grassland	pasture	non-fertilized	NA	Cambisol
HEG44	HAI	grassland	pasture	non-fertilized	NA	Cambisol
HEG45	HAI	grassland	pasture	non-fertilized	NA	Cambisol
HEG46	HAI	grassland	pasture	non-fertilized	NA	Cambisol
HEG47	HAI	grassland	mown pasture	non-fertilized	NA	Cambisol
HEG48	HAI	grassland	mown pasture	non-fertilized	NA	Cambisol
HEG49	HAI	grassland	mown pasture	non-fertilized	NA	Cambisol
HEG50	HAI	grassland	mown pasture	non-fertilized	NA	Cambisol
HEW1	HAI	forest	AKL	NA	spruce	Stagnosol
HEW2	HAI	forest	AKL	NA	spruce	Stagnosol
HEW3	HAI	forest	AKL	NA	spruce	Luvisol
HEW4	HAI	forest	AKL	NA	beech	Luvisol
HEW5	HAI	forest	AKL	NA	beech	Luvisol
HEW6	HAI	forest	AKL	NA	beech	Luvisol
HEW7	HAI	forest	PLW	NA	beech	Luvisol
HEW8	HAI	forest	PLW	NA	beech	Luvisol
HEW9	HAI	forest	PLW	NA	beech	Luvisol
HEW10	HAI	forest	NW	NA	beech	Stagnosol
HEW11	HAI	forest	NW	NA	beech	Luvisol
HEW12	HAI	forest	NW	NA	beech	Luvisol
HEW13	HAI	forest	AKL	NA	spruce	Luvisol
HEW14	HAI	forest	AKL	NA	beech	Luvisol
HEW15	HAI	forest	AKL	NA	beech	Luvisol
HEW16	HAI	forest	AKL	NA	beech	Luvisol
HEW17	HAI	forest	AKL	NA	beech	Luvisol
HEW18	HAI	forest	AKL	NA	beech	Stagnosol
HEW19	HAI	forest	AKL	NA	beech	Luvisol

Table S2 continued.

Site	Exploratory	Land use	Management	Treatment	Tree species	Soil type
HEW20	HAI	forest	AKL	NA	beech	Luvisol
HEW21	HAI	forest	AKL	NA	beech	Luvisol
HEW22	HAI	forest	AKL	NA	beech	Luvisol
HEW23	HAI	forest	AKL	NA	beech	Luvisol
HEW24	HAI	forest	AKL	NA	beech	Luvisol
HEW25	HAI	forest	AKL	NA	beech	Luvisol
HEW26	HAI	forest	PLW	NA	beech	Luvisol
HEW27	HAI	forest	PLW	NA	beech	Luvisol
HEW28	HAI	forest	PLW	NA	beech	Stagnosol
HEW29	HAI	forest	PLW	NA	beech	Luvisol
HEW30	HAI	forest	PLW	NA	beech	Luvisol
HEW31	HAI	forest	PLW	NA	beech	Luvisol
HEW32	HAI	forest	PLW	NA	beech	Luvisol
HEW33	HAI	forest	PLW	NA	beech	Luvisol
HEW34	HAI	forest	NW	NA	beech	Luvisol
HEW35	HAI	forest	NW	NA	beech	Luvisol
HEW36	HAI	forest	NW	NA	beech	Luvisol
HEW37	HAI	forest	NW	NA	beech	Stagnosol
HEW38	HAI	forest	NW	NA	beech	Luvisol
HEW39	HAI	forest	NW	NA	beech	Luvisol
HEW40	HAI	forest	NW	NA	beech	Luvisol
HEW41	HAI	forest	NW	NA	beech	Luvisol
HEW42	HAI	forest	NW	NA	beech	Stagnosol
HEW43	HAI	forest	AKL	NA	beech	Stagnosol
HEW44	HAI	forest	AKL	NA	beech	Stagnosol
HEW45	HAI	forest	AKL	NA	beech	Luvisol
HEW46	HAI	forest	AKL	NA	beech	Luvisol
HEW47	HAI	forest	AKL	NA	beech	Stagnosol
HEW48	HAI	forest	PLW	NA	beech	Stagnosol
HEW49	HAI	forest	PLW	NA	beech	Stagnosol
HEW50	HAI	forest	NW	NA	beech	Stagnosol
AEG1	ALB	grassland	meadow	fertilized	NA	Leptosol
AEG2	ALB	grassland	meadow	fertilized	NA	Leptosol
AEG3	ALB	grassland	meadow	fertilized	NA	Leptosol
AEG4	ALB	grassland	mown pasture	fertilized	NA	Leptosol
AEG5	ALB	grassland	mown pasture	fertilized	NA	Leptosol
AEG6	ALB	grassland	mown pasture	fertilized	NA	Leptosol
AEG7	ALB	grassland	pasture	non-fertilized	NA	Leptosol
AEG8	ALB	grassland	pasture	non-fertilized	NA	Leptosol
AEG9	ALB	grassland	pasture	non-fertilized	NA	Leptosol
AEG10	ALB	grassland	meadow	non-fertilized	NA	Leptosol
AEG11	ALB	grassland	meadow	fertilized	NA	Leptosol
AEG12	ALB	grassland	meadow	fertilized	NA	Leptosol

Table S2 continued.

Site	Exploratory	Land use	Management	Treatment	Tree species	Soil type
AEG13	ALB	grassland	meadow	fertilized	NA	Leptosol
AEG14	ALB	grassland	meadow	fertilized	NA	Leptosol
AEG15	ALB	grassland	meadow	fertilized	NA	Leptosol
AEG16	ALB	grassland	mown pasture	fertilized	NA	Leptosol
AEG17	ALB	grassland	meadow	fertilized	NA	Leptosol
AEG18	ALB	grassland	meadow	fertilized	NA	Leptosol
AEG19	ALB	grassland	mown pasture	fertilized	NA	Leptosol
AEG20	ALB	grassland	pasture	fertilized	NA	Leptosol
AEG21	ALB	grassland	pasture	fertilized	NA	Leptosol
AEG22	ALB	grassland	meadow	non-fertilized	NA	Leptosol
AEG23	ALB	grassland	meadow	non-fertilized	NA	Leptosol
AEG24	ALB	grassland	mown pasture	fertilized	NA	Leptosol
AEG25	ALB	grassland	pasture	non-fertilized	NA	Leptosol
AEG26	ALB	grassland	pasture	non-fertilized	NA	Leptosol
AEG27	ALB	grassland	pasture	non-fertilized	NA	Leptosol
AEG28	ALB	grassland	pasture	non-fertilized	NA	Leptosol
AEG29	ALB	grassland	mown pasture	fertilized	NA	Cambisol
AEG30	ALB	grassland	mown pasture	non-fertilized	NA	Leptosol
AEG31	ALB	grassland	mown pasture	non-fertilized	NA	Leptosol
AEG32	ALB	grassland	pasture	non-fertilized	NA	Leptosol
AEG33	ALB	grassland	pasture	non-fertilized	NA	Leptosol
AEG34	ALB	grassland	pasture	non-fertilized	NA	Leptosol
AEG35	ALB	grassland	meadow	fertilized	NA	Cambisol
AEG36	ALB	grassland	meadow	fertilized	NA	Cambisol
AEG37	ALB	grassland	meadow	fertilized	NA	Cambisol
AEG38	ALB	grassland	meadow	fertilized	NA	Cambisol
AEG39	ALB	grassland	meadow	fertilized	NA	Cambisol
AEG40	ALB	grassland	meadow	fertilized	NA	Cambisol
AEG41	ALB	grassland	meadow	fertilized	NA	Cambisol
AEG42	ALB	grassland	mown pasture	fertilized	NA	Cambisol
AEG43	ALB	grassland	mown pasture	fertilized	NA	Cambisol
AEG44	ALB	grassland	pasture	non-fertilized	NA	Cambisol
AEG45	ALB	grassland	meadow	non-fertilized	NA	Cambisol
AEG46	ALB	grassland	pasture	non-fertilized	NA	Cambisol
AEG47	ALB	grassland	pasture	non-fertilized	NA	Cambisol
AEG48	ALB	grassland	pasture	non-fertilized	NA	Cambisol
AEG49	ALB	grassland	pasture	non-fertilized	NA	Cambisol
AEG50	ALB	grassland	meadow	non-fertilized	NA	Cambisol
AEW1	ALB	forest	AKL	NA	spruce	Cambisol
AEW2	ALB	forest	AKL	NA	spruce	Leptosol
AEW3	ALB	forest	AKL	NA	spruce	Cambisol
AEW4	ALB	forest	AKL	NA	beech	Cambisol
AEW5	ALB	forest	AKL	NA	beech	Cambisol

Table S2 continued.

Site	Exploratory	Land use	Management	Treatment	Tree species	Soil type
AEW6	ALB	forest	AKL	NA	beech	Cambisol
AEW7	ALB	forest	AKL	NA	beech	Leptosol
AEW8	ALB	forest	NW	NA	beech	Cambisol
AEW9	ALB	forest	NW	NA	beech	Leptosol
AEW10	ALB	forest	AKL	NA	spruce	Leptosol
AEW11	ALB	forest	AKL	NA	spruce	Cambisol
AEW12	ALB	forest	AKL	NA	spruce	Cambisol
AEW13	ALB	forest	AKL	NA	spruce	Cambisol
AEW14	ALB	forest	AKL	NA	spruce	Cambisol
AEW15	ALB	forest	AKL	NA	beech	Leptosol
AEW16	ALB	forest	AKL	NA	beech	Cambisol
AEW17	ALB	forest	AKL	NA	beech	Cambisol
AEW18	ALB	forest	AKL	NA	beech	Cambisol
AEW19	ALB	forest	AKL	NA	beech	Cambisol
AEW20	ALB	forest	AKL	NA	beech	Cambisol
AEW21	ALB	forest	AKL	NA	beech	Cambisol
AEW22	ALB	forest	AKL	NA	beech	Cambisol
AEW23	ALB	forest	AKL	NA	beech	Cambisol
AEW24	ALB	forest	AKL	NA	beech	Cambisol
AEW25	ALB	forest	AKL	NA	beech	Cambisol
AEW26	ALB	forest	AKL	NA	beech	Cambisol
AEW27	ALB	forest	AKL	NA	beech	Leptosol
AEW28	ALB	forest	AKL	NA	beech	Cambisol
AEW29	ALB	forest	AKL	NA	beech	Leptosol
AEW30	ALB	forest	AKL	NA	beech	Cambisol
AEW31	ALB	forest	AKL	NA	spruce	Leptosol
AEW32	ALB	forest	AKL	NA	spruce	Leptosol
AEW33	ALB	forest	AKL	NA	spruce	Cambisol
AEW34	ALB	forest	AKL	NA	spruce	Leptosol
AEW35	ALB	forest	AKL	NA	beech	Leptosol
AEW36	ALB	forest	AKL	NA	beech	Leptosol
AEW37	ALB	forest	AKL	NA	beech	Leptosol
AEW38	ALB	forest	AKL	NA	beech	Leptosol
AEW39	ALB	forest	AKL	NA	beech	Leptosol
AEW40	ALB	forest	AKL	NA	beech	Leptosol
AEW41	ALB	forest	AKL	NA	beech	Leptosol
AEW42	ALB	forest	AKL	NA	beech	Leptosol
AEW43	ALB	forest	AKL	NA	beech	Leptosol
AEW44	ALB	forest	AKL	NA	beech	Leptosol
AEW45	ALB	forest	AKL	NA	beech	Cambisol
AEW46	ALB	forest	AKL	NA	beech	Leptosol
AEW47	ALB	forest	AKL	NA	beech	Cambisol
AEW48	ALB	forest	AKL	NA	beech	Leptosol

Table S2 continued.

Site	Exploratory	Land use	Management	Treatment	Tree species	Soil type
AEW49	ALB	forest	NW	NA	beech	Leptosol
AEW50	ALB	forest	AKL	NA	beech	Leptosol

Table S3. Diversity indices and estimates. This table lists the Chao1 richness estimator, Michaelis-Menten-Fit, number of observed species and the diversity indices Shannon (H') and Simpson (D) for all samples, calculated at 5,311 sequences per sample.

Site	Chao 1	Michaelis-Menten-Fit	Number of observed species	Shannon index (H')	Simpson index (D)
SEG1	7094.229	5759.738	2306	10.15	0.998
SEG2	7323.877	6407.304	2509.1	10.43	0.998
SEG3	7670.008	6358.627	2455.1	10.328	0.998
SEG4	4344.132	3546.984	1817.2	9.744	0.997
SEG5	6331.462	5419.245	2255	10.098	0.998
SEG6	8012.599	6909.807	2639.4	10.597	0.999
SEG7	7402.458	6357.476	2452.9	10.302	0.998
SEG8	8856.476	7882.927	2763.1	10.66	0.999
SEG9	7282.602	6309.089	2397.9	10.184	0.998
SEG10	7814.84	6904.029	2612.4	10.561	0.999
SEG11	7428.185	6190.338	2415.3	10.275	0.998
SEG12	7607.704	6768.333	2579.6	10.514	0.998
SEG13	9886.498	8821.943	2798.6	10.543	0.998
SEG14	7839.443	7242.44	2670.7	10.599	0.999
SEG15	4790.82	4041.234	1918.5	9.771	0.997
SEG16	7380.001	6116.738	2431.5	10.344	0.998
SEG17	7205.694	6041.893	2436.6	10.346	0.998
SEG18	8969.864	8021.993	2722.7	10.45	0.997
SEG19	6417.823	5613.748	2303.4	10.186	0.998
SEG20	7431.05	6753.321	2553.4	10.418	0.998
SEG21	6693.213	6013.486	2322	10.038	0.997
SEG22	7185.279	6136.323	2446.7	10.368	0.998
SEG23	7706.474	6573.289	2462.4	10.246	0.997
SEG24	6515.806	5475.427	2288.7	10.204	0.998
SEG25	6834.74	5797.908	2376.1	10.283	0.998
SEG26	6318.051	5430.308	2246.8	10.083	0.998
SEG27	7813.434	6966.592	2602.8	10.461	0.998
SEG28	6296.629	4998.498	2175.4	10.07	0.998
SEG29	5223.306	4294.069	1993.7	9.859	0.997
SEG30	6446.365	5701.008	2260.1	10.026	0.997
SEG31	7369.885	6562.902	2415.4	10.048	0.996
SEG32	7119.621	5901.151	2262.2	9.85	0.995
SEG33	8104.286	7055.753	2595.4	10.413	0.998
SEG34	8648.031	7364.476	2605.6	10.377	0.998
SEG35	6293.491	5155.253	2164.1	9.946	0.997
SEG36	6864.958	5800.902	2290.4	9.992	0.996
SEG37	7005.596	6324.656	2356.7	9.922	0.994
SEG38	6357.294	5261.015	2141.1	9.736	0.995
SEG39	6121.355	5056.049	2126	9.876	0.997

Table S3 continued.

Site	Chao 1	Michaelis-Menten-Fit	Number of observed species	Shannon index (H')	Simpson index (D)
SEG40	8390.666	6856.695	2466.7	10.133	0.996
SEG41	6710	5565.906	2226.6	9.908	0.996
SEG42	7325.056	6109.753	2301.8	9.876	0.995
SEG43	7356.489	6065.728	2297.6	9.91	0.996
SEG44	8049.2	7014.484	2415.5	9.708	0.989
SEG45	6935.908	5888.577	2285.5	9.889	0.995
SEG46	7317.88	6154.615	2279.1	9.772	0.994
SEG47	6935.032	5540.522	2246.4	9.976	0.997
SEG48	7720.198	6759.925	2405	9.986	0.995
SEG49	11338.475	10258.913	3014	10.722	0.998
SEG50	8003.882	6636.919	2332.3	9.745	0.993
SEW1	3226.914	2450.502	1220.7	8.052	0.984
SEW2	4166.034	3098.27	1468.9	8.656	0.991
SEW3	4209.99	3130.77	1474.4	8.475	0.983
SEW4	4225.192	3160.341	1424.6	8.32	0.985
SEW5	3779.391	2879.822	1391.7	8.447	0.989
SEW6	5700.206	4317.668	1783.7	9.085	0.993
SEW7	3841.901	3084.638	1435.7	8.461	0.989
SEW8	4599.68	3697.152	1660.8	9.015	0.993
SEW9	3348.157	2620.492	1292.9	8.376	0.991
SEW10	4100.863	3150.289	1467.8	8.557	0.989
SEW11	3449.996	2678.906	1295.1	8.264	0.988
SEW12	3474.737	2720.213	1288.8	8.162	0.986
SEW13	3472.795	2712.561	1309.5	8.182	0.985
SEW14	4075.648	2930.557	1388.5	8.432	0.989
SEW15	3935.772	3006.317	1463.6	8.738	0.992
SEW16	3544.056	2651.659	1285	8.13	0.983
SEW17	3736.918	2738.553	1294.8	8.159	0.986
SEW18	3557.598	2581.468	1268	8.184	0.987
SEW19	3726.94	2780.928	1335.6	8.402	0.991
SEW20	4292.182	3378.129	1491.5	8.508	0.988
SEW21	3729.98	2865.003	1452.9	8.83	0.993
SEW22	2871.611	2335.542	1189.3	8.071	0.987
SEW23	2839.466	2103.331	1116	8.001	0.988
SEW24	3932.676	3062.104	1496.8	8.841	0.993
SEW25	3656.363	2964.947	1452.4	8.705	0.992
SEW26	4157.099	3225.616	1583	8.986	0.993
SEW27	4897.615	3762.679	1768.9	9.401	0.996
SEW28	5220.853	4155.135	1903.8	9.602	0.996
SEW29	3938.06	3236.631	1608.4	9.164	0.995
SEW30	2298.115	1794.485	1018.5	7.857	0.987
SEW31	3140.168	2311.563	1176.1	8.143	0.989

Table S3 continued.

Site	Chao 1	Michaelis-Menten-Fit	Number of observed species	Shannon index (H')	Simpson index (D)
SEW32	4768.341	3926.295	1649.5	8.782	0.991
SEW33	4246.486	3469.11	1672.8	9.156	0.993
SEW34	3584.837	2969.609	1429.6	8.624	0.991
SEW35	5201.553	4449.65	1885.9	9.377	0.995
SEW36	5224.153	4063.02	1760.5	9.18	0.994
SEW37	5649.108	4595.895	1907.1	9.379	0.995
SEW38	4374.013	3738.485	1741.6	9.258	0.994
SEW39	4091.545	3314.456	1558.2	8.911	0.993
SEW40	5210.123	4088.085	1751.2	9.108	0.993
SEW41	5813.735	4886.967	1884.3	9.212	0.994
SEW42	6230.117	4850.247	1977.8	9.5	0.995
SEW43	6082.497	4975.654	1973.3	9.406	0.994
SEW44	4998.739	4048.157	1691.8	8.934	0.992
SEW45	5486.506	4694.132	1907	9.327	0.994
SEW46	5436.563	4240.365	1861.3	9.405	0.995
SEW47	5665.275	4750.536	2016.1	9.697	0.997
SEW48	5519.121	4634.311	1985.1	9.578	0.995
SEW49	4476.105	3437.385	1575.5	8.91	0.994
SEW50	6161.316	5511.844	2224.3	9.933	0.997
HEG1	7925.276	6993.927	2587.2	10.452	0.998
HEG2	7804.237	7012.619	2615.1	10.522	0.998
HEG3	7725.96	6902.421	2617.9	10.556	0.998
HEG4	8097.095	7090.592	2520.1	10.269	0.998
HEG5	7861.365	7367.644	2703.9	10.635	0.999
HEG6	8407.463	7635.28	2641.7	10.374	0.997
HEG7	6664.923	6020.003	2331.2	10.109	0.997
HEG8	7537.824	6855.176	2616.3	10.533	0.998
HEG9	8789.452	8183.874	2813.6	10.691	0.999
HEG10	7395.908	6644.077	2532.7	10.353	0.997
HEG11	7052.871	5991.567	2340	10.132	0.997
HEG12	7865.953	6446.97	2409.8	10.159	0.997
HEG13	7391.416	6837.799	2533	10.364	0.998
HEG14	7100.277	6019.024	2206.2	9.681	0.995
HEG15	7925.082	6319.681	2364.4	10.111	0.997
HEG16	7025.202	5297.383	2128.3	9.771	0.996
HEG17	6821.349	5733.976	2250.3	9.98	0.997
HEG18	5822.378	4911.61	2085.4	9.771	0.996
HEG19	6858.536	5820.174	2338	10.169	0.998
HEG20	9476.55	8533.684	2760	10.454	0.997
HEG21	7706.758	6306.9	2425.7	10.273	0.998
HEG22	6559.395	5506.084	2214.2	9.932	0.997
HEG23	7703.378	6553.404	2477.4	10.317	0.998

Table S3 continued.

Site	Chao 1	Michaelis-Menten-Fit	Number of observed species	Shannon index (H')	Simpson index (D)
HEG24	8550.141	7556.418	2636	10.425	0.998
HEG25	7443.614	6142.819	2432.9	10.331	0.998
HEG26	6771.309	5536.117	2235.5	10.032	0.997
HEG27	7429.278	6102.886	2349.6	10.117	0.997
HEG28	6773.083	5399.948	2236.3	10.065	0.997
HEG29	6804.499	5993.753	2351.3	10.139	0.997
HEG30	7644.897	6658.264	2484.9	10.292	0.998
HEG31	7739.947	6609.193	2498.5	10.374	0.998
HEG32	8677.593	8224.579	2793.3	10.583	0.998
HEG33	7142.119	6106.662	2374.2	10.113	0.996
HEG34	8046.48	8024.91	2871.1	10.826	0.999
HEG35	7713.075	6550.669	2476.1	10.32	0.998
HEG36	7760.423	6639.811	2526.3	10.414	0.998
HEG37	7849.898	7178.348	2662.1	10.59	0.999
HEG38	8161.055	7387.638	2642.6	10.488	0.998
HEG39	10379.915	9897.247	3035.8	10.843	0.998
HEG40	9410.013	9651.121	3130.1	11.039	0.999
HEG41	7225.035	5895.28	2363.6	10.241	0.998
HEG42	7531.542	7017.655	2604.5	10.484	0.998
HEG43	6787.035	5318.297	2186.4	9.943	0.997
HEG44	7646.666	6062.505	2336.8	10.061	0.997
HEG45	7423.06	6409.286	2464.5	10.338	0.998
HEG46	6910.777	5547.945	2248	10.039	0.997
HEG47	5880.431	4624.264	1967.2	9.594	0.996
HEG48	8119.746	6632.664	2459.6	10.215	0.997
HEG49	5373.033	4289.846	1951.5	9.669	0.996
HEG50	6461.04	5486.6	2247.8	10.045	0.997
HEW1	8650.604	6930.626	2562.3	10.407	0.998
HEW2	8324.796	7151.404	2592.5	10.383	0.997
HEW3	8164.795	7064.625	2473.5	10.095	0.996
HEW4	7944.321	7354.168	2557.7	10.2	0.996
HEW5	7375.756	6264.727	2371.4	10.071	0.997
HEW6	6510.697	5032.176	1983.2	9.459	0.995
HEW7	7365.413	5787.957	2136.3	9.599	0.994
HEW8	8275.611	6699.093	2545.5	10.428	0.998
HEW9	7447.434	5843.373	2200.3	9.774	0.996
HEW10	6833.442	5539.28	2220.8	9.926	0.996
HEW11	7250.425	5469.239	2117.8	9.701	0.996
HEW12	5512.578	4207.001	1782.3	9.175	0.993
HEW13	6361.843	4999.553	2078.7	9.75	0.996
HEW14	6419.51	5137.252	2029	9.423	0.993
HEW15	5386.689	4456.904	1882.5	9.333	0.994

Table S3 continued.

Site	Chao 1	Michaelis-Menten-Fit	Number of observed species	Shannon index (H')	Simpson index (D)
HEW16	6140.342	4792.369	1915.1	9.182	0.99
HEW17	5728.986	4490.939	1813.8	9.094	0.992
HEW18	8156.286	7173.983	2469.5	10.021	0.995
HEW19	7255.033	6351.878	2348.8	9.992	0.996
HEW20	7668.27	6445.536	2395.5	10.068	0.996
HEW21	8042.853	6639.189	2394.7	10.02	0.996
HEW22	9343.535	8263.172	2604.2	10.139	0.996
HEW23	11772.515	10323.203	2889.2	10.446	0.997
HEW24	12600.362	10058.618	2920.5	10.617	0.998
HEW25	12625.689	10046.659	2800.8	10.309	0.996
HEW26	10296.499	8582.787	2664.6	10.299	0.997
HEW27	10800.155	9476.954	2966.1	10.79	0.999
HEW28	13704.666	12792.52	3368.4	11.143	0.999
HEW29	7564.013	5891.861	2141.3	9.649	0.996
HEW30	7986.106	6476.243	2242	9.731	0.995
HEW31	8147.447	6557.371	2298.4	9.88	0.996
HEW32	9662.948	7766.849	2500	10.051	0.996
HEW33	11004.471	9660.192	2839.9	10.484	0.997
HEW34	11096.224	10072.176	2893	10.496	0.997
HEW35	9044.84	7056.686	2450.4	10.093	0.997
HEW36	11074.79	9002.528	2792.7	10.462	0.997
HEW37	10125.4	8410.339	2668.9	10.314	0.997
HEW38	12826.544	10406.134	3010.1	10.747	0.998
HEW39	8722.412	7115.261	2399.7	9.909	0.995
HEW40	14432.655	13698.182	3408.8	11.118	0.999
HEW41	11686.629	9831.282	2875.7	10.508	0.997
HEW42	10877.836	8983.633	2669.1	10.228	0.997
HEW43	12556.006	10624.598	3012.2	10.658	0.997
HEW44	15267.118	14225.105	3495.6	11.233	0.999
HEW45	8774.148	7747.68	2713.2	10.587	0.998
HEW46	8541.199	6608.822	2311.5	9.87	0.996
HEW47	13582.314	11391.486	3098.6	10.767	0.998
HEW48	10442.486	8944.448	2706.5	10.294	0.996
HEW49	9315.865	7726.909	2506.8	10.042	0.996
HEW50	8394.801	7022.75	2425.4	10.034	0.997
AEG1	6509.742	5603.182	2328.9	10.236	0.998
AEG2	6186.869	5312.725	2248.6	10.134	0.998
AEG3	6056.487	4978.924	2172	10.005	0.997
AEG4	6852.583	5879.876	2390.9	10.297	0.998
AEG5	7412.708	6206.475	2407.2	10.222	0.998
AEG6	7534.877	6005.639	2302.9	10.001	0.997
AEG7	6290.972	5205.158	2221.4	10.118	0.998

Table S3 continued.

Site	Chao 1	Michaelis-Menten-Fit	Number of observed species	Shannon index (H')	Simpson index (D)
AEG8	6766.84	5397.583	2184.9	9.893	0.996
AEG9	5570.335	4709.748	2044.7	9.698	0.995
AEG10	6010.696	4891.221	2096.1	9.799	0.996
AEG11	6250.92	5591.978	2335.5	10.225	0.998
AEG12	6315.959	5157.856	2207.4	10.084	0.998
AEG13	5726.15	4854.779	2092.7	9.839	0.997
AEG14	6376.177	5292.335	2200.2	10.017	0.997
AEG15	6199.153	5196.339	2197.9	10.011	0.997
AEG16	6951.524	5663.081	2279	10.088	0.997
AEG17	6369.211	5342.349	2220.2	10.024	0.997
AEG18	5440.358	4446.025	1994.4	9.749	0.997
AEG19	5926.76	5006.72	2110.4	9.836	0.996
AEG20	6654.157	5896.851	2319.4	10.095	0.997
AEG21	6319.584	5243.971	2201.1	9.995	0.997
AEG22	6813.146	5515.524	2259.6	10.034	0.997
AEG23	6218.697	5234.001	2167.8	9.914	0.997
AEG24	6213.207	4944.084	2099	9.788	0.996
AEG25	6407.637	5356.046	2194.2	9.934	0.997
AEG26	6780.735	5874.224	2327.1	10.08	0.997
AEG27	6866.719	5824.318	2293.8	9.976	0.996
AEG28	6319.787	5009.022	2122.8	9.91	0.997
AEG29	7574.405	6261.025	2312.6	9.893	0.995
AEG30	6415.742	5119.894	2185.1	10.038	0.998
AEG31	6989.857	5677.971	2254.7	9.999	0.997
AEG32	8228.713	7239.791	2601.4	10.371	0.997
AEG33	6863.883	5576.393	2230.5	9.865	0.995
AEG34	5565.443	4585.045	2038.4	9.848	0.997
AEG35	7004.054	5774.322	2275.3	9.942	0.995
AEG36	6491.113	5181.385	2187.8	9.957	0.996
AEG37	6593.61	5162.588	2171.6	9.954	0.997
AEG38	5893.134	5147.187	2163.4	9.915	0.996
AEG39	6739.306	5599.151	2295	10.156	0.998
AEG40	5661.46	4331.422	1979.5	9.798	0.997
AEG41	6187.209	4941.962	2160.6	10.042	0.998
AEG42	6900.878	5677.101	2225.2	9.948	0.997
AEG43	7545.519	6563.508	2501.5	10.37	0.998
AEG44	7051.659	6219.606	2408.5	10.227	0.998
AEG45	5847.468	4874.353	2100.9	9.793	0.996
AEG46	6644.454	5682.535	2308	10.144	0.998
AEG47	7263.009	6062.201	2379.4	10.211	0.998
AEG48	6700.199	5547.513	2278.7	10.146	0.998
AEG49	6721.956	5534.606	2311.9	10.205	0.998

Table S3 continued.

Site	Chao 1	Michaelis-Menten-Fit	Number of observed species	Shannon index (H')	Simpson index (D)
AEG50	7271.138	6411.706	2522.1	10.446	0.998
AEW1	4147.685	2946.909	1359	8.319	0.989
AEW2	5779.795	4226.617	1739.8	8.967	0.992
AEW3	6263.087	5258.342	2203.8	10.005	0.997
AEW4	4734.699	3756.306	1742.3	9.183	0.992
AEW5	5103.505	3793.186	1733.8	9.132	0.992
AEW6	4516.37	3518.038	1676.3	9.153	0.994
AEW7	4061.702	2994.617	1455.6	8.621	0.99
AEW8	4576.867	3674.305	1717.1	9.15	0.993
AEW9	3454.318	2645.924	1370.8	8.697	0.993
AEW10	3095.173	2460.067	1314	8.533	0.99
AEW11	2875.121	2084.505	1080.8	7.852	0.986
AEW12	4221.973	3229.57	1515.3	8.696	0.99
AEW13	3173.415	2363.999	1237.2	8.274	0.989
AEW14	5107.693	4069.705	1866.5	9.446	0.994
AEW15	4776.078	3752.416	1742	9.221	0.994
AEW16	5033.195	4109.862	1798.9	9.07	0.989
AEW17	4462.391	3611.231	1741.6	9.318	0.994
AEW18	7298.052	6401.647	2385.4	10.055	0.996
AEW19	3619.005	2951.212	1436.4	8.588	0.99
AEW20	6633.477	5381.162	2247.3	10.082	0.997
AEW21	7480.323	6446.085	2445.4	10.243	0.997
AEW22	6249.586	5098.035	2136.6	9.79	0.995
AEW23	5499.94	4448.112	1930.5	9.411	0.993
AEW24	6906.674	5617.582	2182.9	9.741	0.995
AEW25	5053.817	4155.117	1748	9.014	0.991
AEW26	6081.067	4837.371	2049.3	9.657	0.995
AEW27	4800.17	3765.774	1739.1	9.22	0.994
AEW28	6394.175	5497.115	2159.1	9.723	0.995
AEW29	4925.566	3884.101	1769.3	9.214	0.993
AEW30	6515.459	5681.403	2284	10.039	0.997
AEW31	5329.834	4429.401	1999.1	9.738	0.996
AEW32	4969.466	3951.603	1839.4	9.521	0.996
AEW33	3898.764	3179.409	1580	8.95	0.991
AEW34	4613.363	3561.339	1719.5	9.291	0.994
AEW35	7514.192	6216.002	2380.6	10.098	0.996
AEW36	8877.86	7188.271	2539.6	10.218	0.996
AEW37	8667.86	7639.726	2638.3	10.352	0.997
AEW38	6170.329	4897.242	2035.3	9.573	0.994
AEW39	9569.422	8952.119	2808.7	10.45	0.996
AEW40	5962.302	4797.357	2013.4	9.561	0.994
AEW41	7828.788	6527.176	2387.2	9.992	0.996

Table S3 continued.

Site	Chao 1	Michaelis-Menten-Fit	Number of observed species	Shannon index (H')	Simpson index (D)
AEW42	9075.141	8033.014	2794	10.695	0.999
AEW43	7029.852	5402.884	2120.8	9.6	0.993
AEW44	7346.448	5665.453	2222.3	9.823	0.995
AEW45	6178.309	4985.523	2061.8	9.59	0.994
AEW46	8785.3	7755.439	2700.1	10.497	0.998
AEW47	5896.774	4663.848	1956.5	9.414	0.993
AEW48	7015.329	5753.076	2356.8	10.229	0.998
AEW49	8413.35	7781.44	2771.2	10.639	0.998
AEW50	7085.739	5720.121	2214.6	9.788	0.995

Table S4. Shannon indices (H') for the different management regimes per exploratory and land use. Letters indicate significant ($P < 0.05$) differences between management regimes according to Holm's test (mean ± SD).

Exploratory	Land use	Management	Fertilization / tree species	Mean H'
Schwäbische Alb	Grassland	meadow	non-fertilized	10.00 ± 0.3
			fertilized	10.00 ± 0.1
		pasture	non-fertilized	10.04 ± 0.2
			fertilized	10.05 ± 0.1
		mown pasture	non-fertilized	10.02 ± 0.0
			fertilized	10.05 ± 0.2
	Forest	AKL	beech	9.67 ± 0.5[a]
			spruce	8.97 ± 0.7[b]
		NW	beech	9.50 ± 1.0[ab]
Hainich-Dün	Grassland	meadow	fertilized	10.31 ± 0.2
		pasture	non-fertilized	10.30 ± 0.3
			fertilized	10.11 ± 0.0
		mown pasture	non-fertilized	10.09 ± 0.3
			fertilized	10.30 ± 0.3
	forest	AKL	beech	10.07 ± 0.6
			spruce	10.16 ± 0.3
		NW	beech	10.21 ± 0.5
		PLW	beech	10.17 ± 0.5
Schorfheide-Chorin	grassland	meadow	non-fertilized	10.14 ± 0.2
			fertilized	10.40 ± 0.2
		pasture	non-fertilized	10.12 ± 0.3
			fertilized	9.90 ± 0.2
		mown pasture	non-fertilized	10.10 ± 0.4
			fertilized	10.25 ± 0.3
	Forest	AKL	beech	9.24 ± 0.3[a]
			oak	8.84 ± 0.6[ab]
			pine	8.41 ± 0.3[b]
		NW	beech	9.02 ± 0.6[ab]

Table S5. Key enzymes involved in cycling of carbon, nitrogen, sulfur and phosphorus. Significance tested by applying the Mann-Whitney test. The land use in which the abundance of the key genes was significantly higher (P<0.05) is indicated.

Cycle	Pathway	Key Enzyme/s (KO)	Upregulated in (P <0.05)	Reference
Carbon cycle	Calvin Benson cycle	RuBisCO (K01601, K01602)	forest	[1]
	Reductive citric acid cycle	Fumarate reductase (K00239, K00240)	grassland	[1]
		Ferredoxin-dependent 2-oxoglutarare synthase		[1]
		(K00174, K00175)	grassland	
		(K00176, K00177)	forest	
		ATP citrate lyase (K15230, K15231)	n.a.	[1]
	Wood-Ljungdahl pathway	Carbon monoxide dehydrogenase		[1]
		(K00192)	forest	
		(K00198)	grassland	
	Hydroxypropionate Bicycle	Biotin-dependent acetyl-CoA/propionyl-CoA carboxylase (K01964, K01965, K01966, K11263)	grassland	[1]
	Cellulose breakdown	Endoglucanase (K01179)	grassland	
		Endo-1,3(4)-beta-glucanase (K01180)	grassland	
		Cellulose 1,4-beta-cellobiosidase (K01225)	grassland	
		Beta-glucuronidase (K01195)	forest	
	Xylan breakdown	Endo-1,4-beta-xylanase (K01181)	grassland	
		Xylan 1,4-beta xylosidase (K01198)	forest	
	Chitin breakdown	Chitinase (K01183)	grassland	
	Lignin breakdown	Biphenyl dioxygenase (K08689, K15750)	grassland	[2]
		Dihydrodiol dehydrogenase ((K14582)	forest	[2]

Table S5 continued.

Cycle	Pathway	Key Enzyme/s (KO)	Upregulated in (P <0.05)	Reference
		Phenylcatechol dioxygenase (K03381)	grassland	[2]
	PAH breakdown	Catechol 2,3-dioxygenase (K00446)	forest	[3]
		Naphthalene dioxygenase (K14579, K14580)	grassland	[3]
Nitrogen cycle	Denitrification			
		Nitrate reductase, nirS (K15864)	grassland	
		nitrite reductase, nirK (K00368)	grassland	
	Nitrification	Ammonia moonooxygenase, amoABC (K10944, K10945, K10946)	grassland	[4]
	Dissimilatory nitrate reduction	Nitrate reductase, narG (K00370)	grassland	
		Nitrite reductase, nirB (K00362)	grassland	
	Assimilatory nitrate reduction	Ferredoxin-nitrate reductase, narB (K00367)	forest	
		Ferredoxin-nitrite reductase, nirA (K00366)	forest	
	Nitrogen fixation	Nitrogenase, nifH (K02588)	forest	
	Anammox	Hydroxylamine oxidase (K10535)	grassland	
		Urease (K01428, K01429, K01430)	grassland	
Sulfur cycle		Adenylylsulfate reductase, aprAB		[5]
	Dissimilatory sulfate reduction	(K00394)	grassland	
		(K00395)	forest	
	Assimilatory sulfate reduction	Adenylylsulfate kinase, cysC (K00860)	forest	

Table S5 continued.

Cycle	Pathway	Key Enzyme/s (KO)	Upregulated in (P <0.05)	Reference
		Phosphoadenosine phosphosulfate reductase, cysH (K00390)	forest	
Methane cycle	Aerobic methane oxidation	Methanol dehydrogenase /cytochrome c (K14028, K14029)	forest	[5]
Phosphorus cycle		acid phosphatase (K01078, K01093, K03788, K09474)	forest	
		alkaline phosphatase (K01077, K01113)	grassland	

References

1. Berg, I.A. Ecological aspects of the distribution of different autotrophic CO2 fixation pathways. *Appl Environ Microbiol.* **77**, 1925-1936 (2011).
2. Zimmermann, W. Degradation of lignin by bacteria. *J Biotechnol.* **13**, 119-130 (1990).
3. Meyer, S., Moser, R., Neef, A., Stahl, U. & Kämpfer, P. Differential detection of key enzymes of polyaromatic-hydrocarbon-degrading bacteria using PCR and gene probes. *Microbiology*. **145**, 1731-1741 (1999).
4. Rotthauwe, J.H., Witzel, K.P. & Liesack, W. The ammonia monooxygenase strucural gene amoA as a functional marker: molecular fine-scale analysis of natural ammonia-oxidizing populations. *Appl Environ Microbiol.* **63**, 4704-4712 (1997).
5. Wrede, C., Krukenberg, V., Dreier, A., Reitner, J., Heller, C. & Hoppert, M. Detection of metabolic key enzymes of methane turnover processes in cold seep microbial biofilms. *Geomicrobiol J*. **30**, 214-227 (2012).
